# Engineering Separated Dual O_2_ Reduction Cores into One Polymer Framework for Boosting Hydrogen Peroxide Production

**DOI:** 10.1002/advs.202508553

**Published:** 2025-06-29

**Authors:** Mengmeng Fu, Jialun He, Yingguo Li, Jiacheng Liu, Chensheng Wang, Jiayi Lu, Chaoyang Ma, Danfeng Jiang, Xiao Chen, Chao Yu

**Affiliations:** ^1^ School of Environmental and Chemical Engineering Jiangsu University of Science and Technology Zhenjiang Jiangsu 212003 P. R. China

**Keywords:** covalent organic polymers, hydrogen peroxide, microreactor, oxygen reduction reaction, self‐marketing & cooperation

## Abstract

Photocatalytic hydrogen peroxide (H_2_O_2_) production through the oxygen reduction reaction (ORR) pathway has emerged as a promising sustainable alternative. However, a significant challenge in this field lies in the development of highly efficient photocatalysts capable of achieving high‐concentration H_2_O_2_ production. Here the rational design of two O_2_ reduction cores into one polymer framework is showcased for synergistically facilitating H_2_O_2_ production via the “self‐marketing & cooperation” strategy. Photoactive units of pyrrolo[3,2‐*b*] pyrrole and porphyrin are successfully settled in covalent organic polymers (COPs) through polycyclizations of the relevant aldehydes, anilines, and butane‐2,3‐dione. Two orderly separated active sites not only involve each of them in the oxygen reduction reaction but also mutually promote the production of H_2_O_2_, which is demonstrated by electron spin resonance experiments, in situ diffuse reflectance infrared Fourier transform spectroscopy, and a series of control experiments. Remarkably, PP‐COP‐**4** delivers an outstanding H_2_O_2_ concentration of 16.2 mm in a continuous‐flow system, demonstrating its strong potential for scalable, solar‐driven production of commercial‐grade H_2_O_2_.

## Graphical abstract

Here the rational design of two O_2_ reduction cores into one polymer framework is showcased for synergistically facilitating H_2_O_2_ production via the “selfmarketing & cooperation” strategy. Photoactive units of pyrrolo[3,2‐*b*]pyrrole and porphyrin are successfully settled in covalent organic polymers (COPs) through polycyclizations of the relevant aldehydes, anilines, and butane‐2,3‐dione. PP‐COP‐**4** delivers an outstanding H_2_O_2_ concentration of 16.2 mm in a continuous‐flow system, demonstrating its strong potential for scalable, solar‐driven production of commercial‐grade H_2_O_2_.

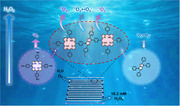

## 1 Introduction

Hydrogen peroxide is a versatile and environmentally friendly oxidant with extensive applications in chemical synthesis,^[^ ^1^ ^]^ medical sterilization^[^ ^2^ ^]^ and energy conversion.^[^ ^3^ ^]^ However, the industrial‐scale production of H_2_O_2_ predominantly relies on the anthraquinone oxidation process, which suffers from several inherent drawbacks, including serious waste and high energy consumption.^[^ ^4^ ^]^ Thus, the development of efficient, clean, and sustainable H_2_O_2_ production methods is crucial to addressing these challenges. Photocatalytic H_2_O_2_ production via the ORR pathway has emerged as an attractive sustainable alternative, owing to the inherent advantages of photocatalysis, including renewable energy utilization, mild reaction conditions, and environmental benignity.^[^ ^5^ ^]^ Several inorganic and organic semiconductors have been developed to meet the energy requirements for photocatalytic production of H_2_O_2_.^[^ ^6^ ^]^ Compared to inorganic semiconductors, organic semiconductors including covalent organic frameworks/polymers (COFs/COPs) feature precisely tunable energy band structures, good environmental compatibility, and processability, which have promoted them as promising candidates for sustainable H_2_O_2_ generation.^[^ ^7^ ^]^

**Figure 1:**
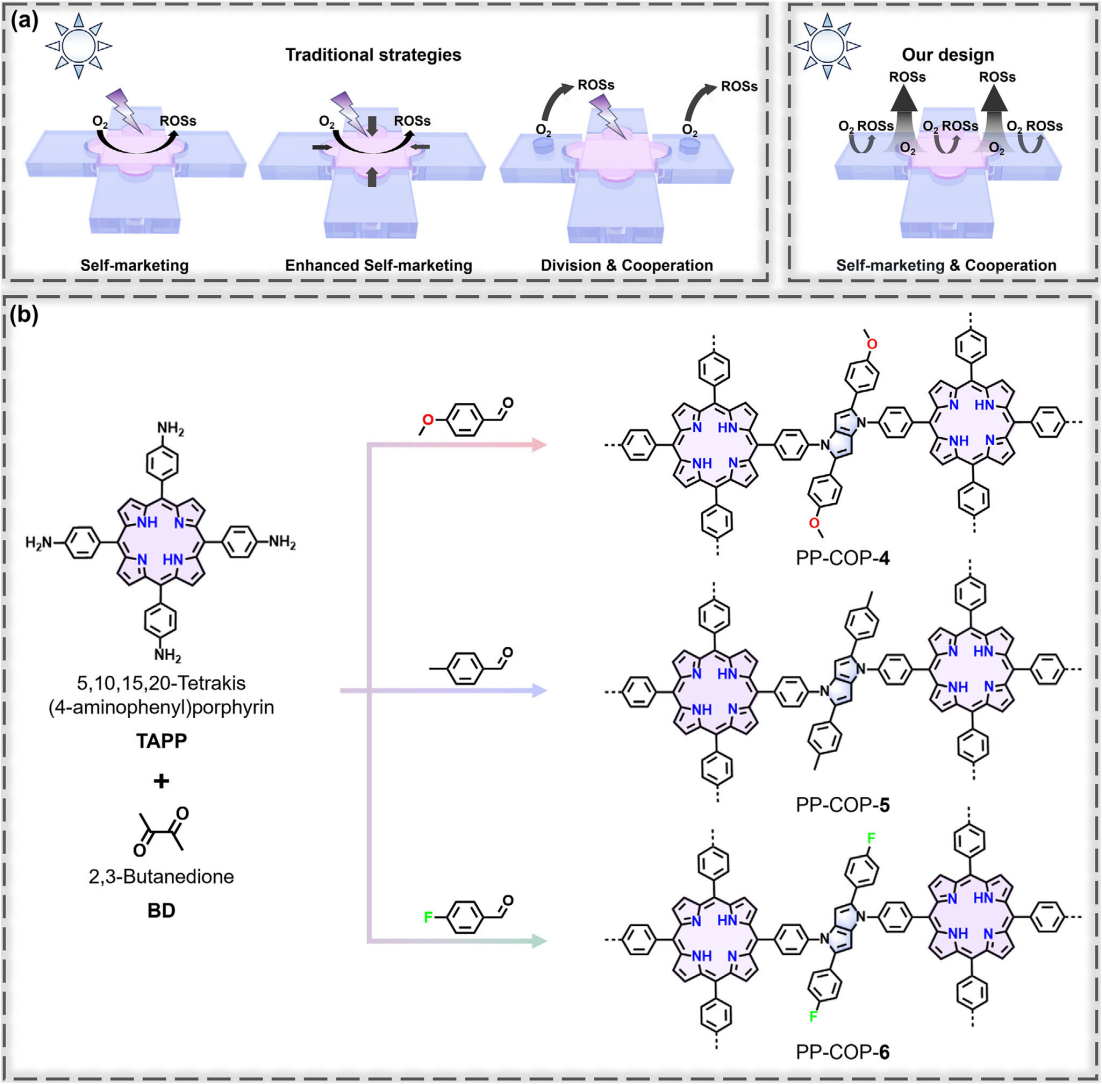
a) Traditional strategies and our design for the photosynthesis of ROSs. b) Schematic synthesis of PP‐COPs **4–6**.

In previous studies, triazine,^[^ ^8^ ^]^ triphenyltriazine,^[^ ^9^ ^]^ benzobisthiazole,^[^ ^10^ ^]^ porphyrin,^[^ ^11^ ^]^ and other photoactive moieties^[^ ^12^ ^]^ have been widely used in the fabrication of COFs/COPs to enhance the yield of H_2_O_2_ through the well‐designed strategies of “self‐marketing”, “enhanced self‐marketing” and “division & cooperation” (**Figure** **1**a).^[^ ^13^ ^]^ However, we found that employing full active units in the COFs/COPs for independent ORR, while harnessing their synergistic effects to enhance the effective utilization of photocatalytic active sites, represents a more efficient yet challenging approach. We designate this innovative strategy as “self‐marketing & cooperation”. We envisage that the two oxygen reduction centers of pyrrolo[3,2‐*b*]pyrrole and porphyrin act as built‐in “mini‐factories”, each capable of independently producing reactive oxygen species (ROS) of ^1^O_2_ and •O_2_^−^, achieving “self‐marketing”. Additionally, the synergistic interaction between these two independently functioning moieties within the polymer framework enables the activation of multiple ORR active sites under illumination, thereby enhancing the utilization efficiency of photocatalytic centers and achieving effective cooperative catalysis.

In this work, three PP‐COPs bearing pyrrolo[3,2‐*b*]pyrrole and porphyrin functionalities were constructed through polycyclizations of the relevant aldehydes, anilines, and butane‐2,3‐dione. The successful assemble of PP‐COPs **4–6** was verified by Fourier transform infrared (FT‐IR) spectra, ^13^C cross‐polarization magic angle spinning solid‐state (^13^C CP‐MAS) NMR spectroscopy and X‐ray photoelectron spectroscopy (XPS). Compared with its precursors, the constructed PP‐COPs exhibited a significant enhancement of the H_2_O_2_ production capacity. This can be attributed to the effect of the “self‐marketing & cooperation” of two O_2_ reduction cores as evidenced by experimental studies and theoretical simulations. Additionally, the microreactor technology was introduced in the production of H_2_O_2_, enabling continuous H_2_O_2_ generation in 9 h with a concentration of 14.8 mm, surpassing the most reported polymer photocatalysts for photosynthesis of H_2_O_2_. This also highlights the advantages of integrating rationally designed dual O_2_ reduction catalysts with advanced microreactor systems, offering a promising direction for future research in sustainable H_2_O_2_ production.

## 2 Results and Discussion

**Figure 2:**
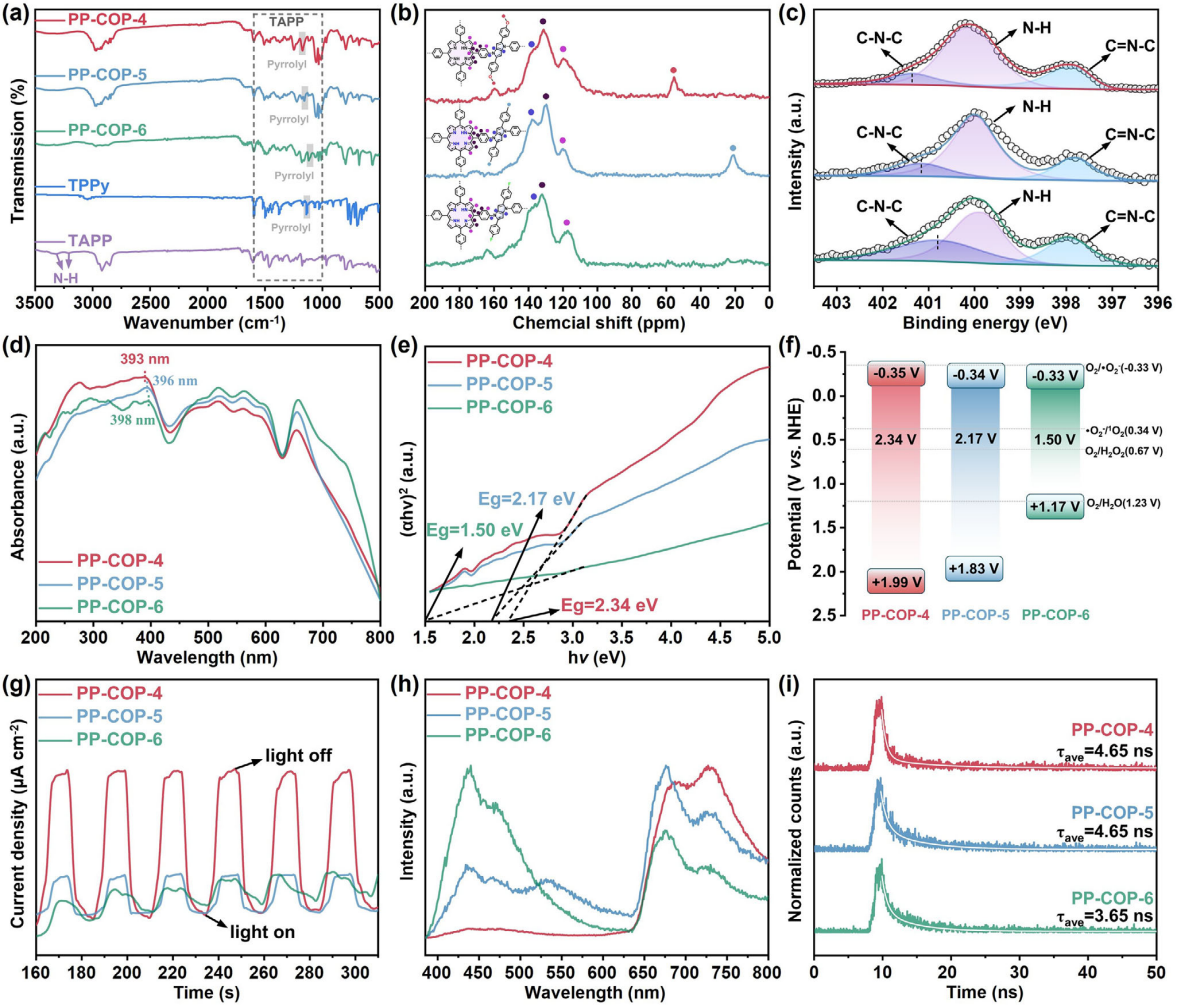
a) FTIR spectra of PP‐COPs **4–6**. b) ^13^C NMR spectra of PP‐COPs **4–6**. c) XPS analysis of N 1s for PP‐COPs **4–6**. d) UV–vis spectra of PP‐COPs **4–6**. e) Characterization of the energy band gaps of PP‐COPs **4–6** obtained from UV–vis spectra. f) Schematic band structure diagram of PP‐COPs **4–6**. g) Transient photocurrents of PP‐COPs **4–6**. h) Steady‐state PL spectra of PP‐COPs **4–6**. i) Time‐resolved photoluminescent decay plots of PP‐COPs **4–6**.

Prior to the synthesis of PP‐COPs **4–6**, the model compound of 1,2,4,5‐tetraphenyl‐1,4‐dihydropyrrolo[3,2‐*b*]pyrrole (TPPy) was prepared via a condensation reaction of benzaldehyde, aniline, and butanedione in a 1:4:2 molar ratio in glacial acetic acid under reflux for 24 h. The formation of TTPy was evidenced by Fourier transform infrared (FT‐IR) spectra (Figure S1, Supporting Information).^[^ ^14^ ^]^ Subsequently, PP‐COPs **4–6** were synthesized via a similar reaction condition, with 5,10,15,20‐tetra(4‐aminophenyl)‐porphyrin (TAPP) serving as the precursor, reacting with three different benzaldehyde monomers including p‐methoxybenzaldehyde, p‐methylbenzaldehyde, and p‐fluorobenzaldehyde, as well as butane‐2,3‐dione, in a molar ratio of 1:4:2 in CH_3_COOH for 3 days (Figure 1b). In the FT‐IR spectra (**Figure** **2**a), the C═O stretching vibration peaks at 1676 cm^−1^ for the benzaldehyde derivatives and 1714 cm^−1^ for 2,3‐butanedione almost disappeared, indicating that these monomers have been consumed. Similarly, the weakening of N─H peaks of TAPP at 3207 and 3318 cm^−1^ strongly supports that TAPP was involved in the polymerization.^[^ ^15^ ^]^ Additionally, new peaks at 1170, 1150, and 1118 cm^−1^ were observed and attributed to the characteristic peaks of the pyrrolyl groups.^[^ ^16^ ^]^ Furthermore, the distinctive peaks associated with the porphyrin moieties were observed at 1010, 1371, and 1596 cm^−1^.^[^ ^17^ ^]^ These results indicate that the successful synthesis of the three PP‐COPs.

The ^13^C cross‐polarization magic angle spinning solid‐state (^13^C CP‐MAS) NMR spectroscopy successfully identified various carbon environments within the polymer samples. Specifically, the aromatic carbon atoms, including those with C═C and C═C─N bonds, as well as the C═C carbon atoms in the pyrrolo[3,2‐*b*]pyrrole structure, exhibited signals in the range of 106.2 to 158.5 ppm (Figure 2b).^[^ ^18^ ^]^ Additionally, the peak at 55.3 ppm can be attributed to the methoxy C─H carbon in PP‐COP‐**4**, while the peak at 21.1 ppm corresponds to the C‐H carbon of the methyl group in PP‐COP‐**5** (Figure 2b).

The comprehensive analysis of XPS spectra confirmed the presence of C, N, O, and F elements (Figure S2, Supporting Information), which is consistent with the predicted composition of these COPs. In the high‐resolution N 1s spectra of PP‐COPs **4**–**6** (Figure 2c), the distinct peaks at ≈397.9 eV were identified as the C═N─C bonds in the pyrrole rings, the peaks at ≈399.9 eV represented the N─H bonds in the porphyrin rings. The peaks at 401.38, 401.11, and 400.85 eV were attributed to the C─N─C bonds in the pyrrolo[3,2‐*b*]pyrrole of PP‐COPs **4**–**6**, respectively.^[^ ^14^, ^19^ ^]^ Notably, the gradual blue shift in the N 1s binding energies of the pyrrolo[3,2‐*b*]pyrrole groups reflects the electron‐withdrawing influence of the substituents, which delocalizes electron density and reduces the effective nuclear charge on nitrogen. Additionally, in the high‐resolution O 1s spectrum of PP‐COP‐**4**, the deconvoluted peak at 531.38 eV was observed, which indicates the presence of C─O─C bonds in the structure (Figure S3, Supporting Information). Similarly, the deconvoluted peak at 687.2 eV in the high‐resolution F 1s spectrum of PP‐COP‐**6** can be attributed to the C─F bonds (Figure S4, Supporting Information). These results further demonstrate the successful synthesis of PP‐COPs and enable the fine‐tuning of their electronic structure.

Scanning electron microscopy (SEM) images revealed that all three PP‐COPs exhibit regular spherical morphologies with micrometer‐scale particle sizes (Figure S5, Supporting Information). Energy dispersive spectroscopy (EDS) mapping confirmed the uniform distribution of C, O, N, and F elements within the polymer materials (Figure S6, Supporting Information). The thermal stabilities of the PP‐COPs were evaluated by using thermal gravimetric analysis (TGA). It was found that three COPs are stable up to ≈250 °C (Figure S7, Supporting Information).^[^ ^20^ ^]^ To evaluate the chemical stability of PP‐COPs, we immersed them for one week in various solvents, including dichloromethane, acetone, ethanol, and tetrahydrofuran. The FT‐IR spectra analysis revealed that the characteristic peaks of these polymers remained essentially unchanged after immersion, confirming their excellent stability (Figure S8, Supporting Information). The Brunauer‐Emmett‐Teller (BET) surface areas of PP‐COPs **4–6** were determined to be 15.32, 32.35, and 74.14 m^2^g^−1^, respectively, which indicated that these PP‐COPs can be classified as nonporous polymer materials (Figure S9, Supporting Information).^[^ ^21^ ^]^ This result is consistent with the theoretical structural simulation (Figure S10, Supporting Information). Water contact angle measurements demonstrated that PP‐COPs possess hydrophilic properties, as evidenced by water contact angles ranging from 59.8–77.9° (Figure S11, Supporting Information). This characteristic is particularly advantageous for catalytic applications in aqueous media, as it promotes effective interfacial interactions between the catalyst and substrates.

The superior photochemical activities of porphyrin and pyrrolo[3,2‐*b*]pyrrole moieties of PP‐COPs promoted us to explore their utilization as a promising photocatalyst. The optoelectronic properties of PP‐COPs **4–6** were studied as a start for the photocatalytic study. As shown in Figure 2d, three PP‐COPs displayed exceptional light absorption capabilities across the ultraviolet to near‐infrared region. Notably, PP‐COPs **4–6** exhibited three distinct Q absorption bands within the 500 to 700 nm visible light range.^[^ ^11^, ^12^, ^22^ ^]^ The band gaps (*E*g) of PP‐COPs **4–6** were determined to be 2.34, 2.17 and 1.50 eV via Tauc plots (Figure 2e), respectively. Interestingly, while the three polymers show very similar light absorption properties, their calculated band gaps display significant variation. This can be attributed to the side‐chain groups of methoxy, methyl, and fluorine having a significant impact on the electronic structure of the polymers, while the similar light absorption properties reflect the dominant role of the main structure (porphyrin moieties) in visible light absorption.^[^ ^23^ ^]^ Mott‐Schottky analysis revealed that the conduction band (CB) potentials of PP‐COPs **4–6** are positioned at −0.35, −0.34, and −0.33 V (vs NHE, Figure 2f; Figure S12, Supporting Information), respectively, while their valence band (VB) energies were calculated as +1.99, +1.83, and +1.42 V (vs NHE), respectively. These results align well with the XPS valence band spectra (Figure S13, Supporting Information). Such favorable band edge positions suggest that these PP‐COPs are thermodynamically capable of driving photocatalytic H_2_O_2_ production via the oxygen reduction pathway.^[^ ^24^ ^]^

The charge dynamics of the PP‐COPs were investigated using transient photocurrent measurements, electrochemical impedance spectroscopy (EIS), and time‐resolved fluorescence decay spectroscopy. As depicted in Figure 2g and Figure S14 (Supporting Information), PP‐COP‐**4** exhibits the highest photocurrent density and the lowest charge transfer resistance, indicating superior charge separation efficiency and enhanced charge transport capabilities.^[^ ^25^ ^]^ Steady‐state photoluminescence (PL) tests of PP‐COPs **4–6** were performed at the excitation of 365 nm (Figure 2h). Two distinct fluorescence emission peaks were observed at ≈440 and 720 nm, corresponding to the emission of pyrrolo[3,2‐*b*]pyrrole and porphyrin chromophores, respectively.^[^ ^14^, ^26^ ^]^ This assignment is further corroborated by the emission profiles of the monomers TAPP and 1,2,4,5‐tetraphenyl‐1,4‐dihydropyrrolo[3,2‐*b*]pyrrole(TPPy), which exhibit characteristic peaks at 432 and 720 nm, respectively (Figure S15, Supporting Information). Time‐resolved PL spectroscopy revealed the average fluorescence lifetimes of PP‐COP‐**4** and PP‐COP‐**5** (4.65 ns) were longer than that of PP‐COP‐**6** (3.65 ns) at an emission of 440 nm (Figure 2i). Similarly, at 720 nm, PP‐COP‐**4** displayed the longest lifetime (0.76 ns), followed by PP‐COP‐**5** (0.66 ns) and PP‐COP‐**6** (0.62 ns) (Figure S16, Supporting Information). These results confirm the successful incorporation of both pyrrolo[3,2‐*b*]pyrrole and porphyrin units into the polymer framework while preserving their distinct optical properties.

**Figure 3:**
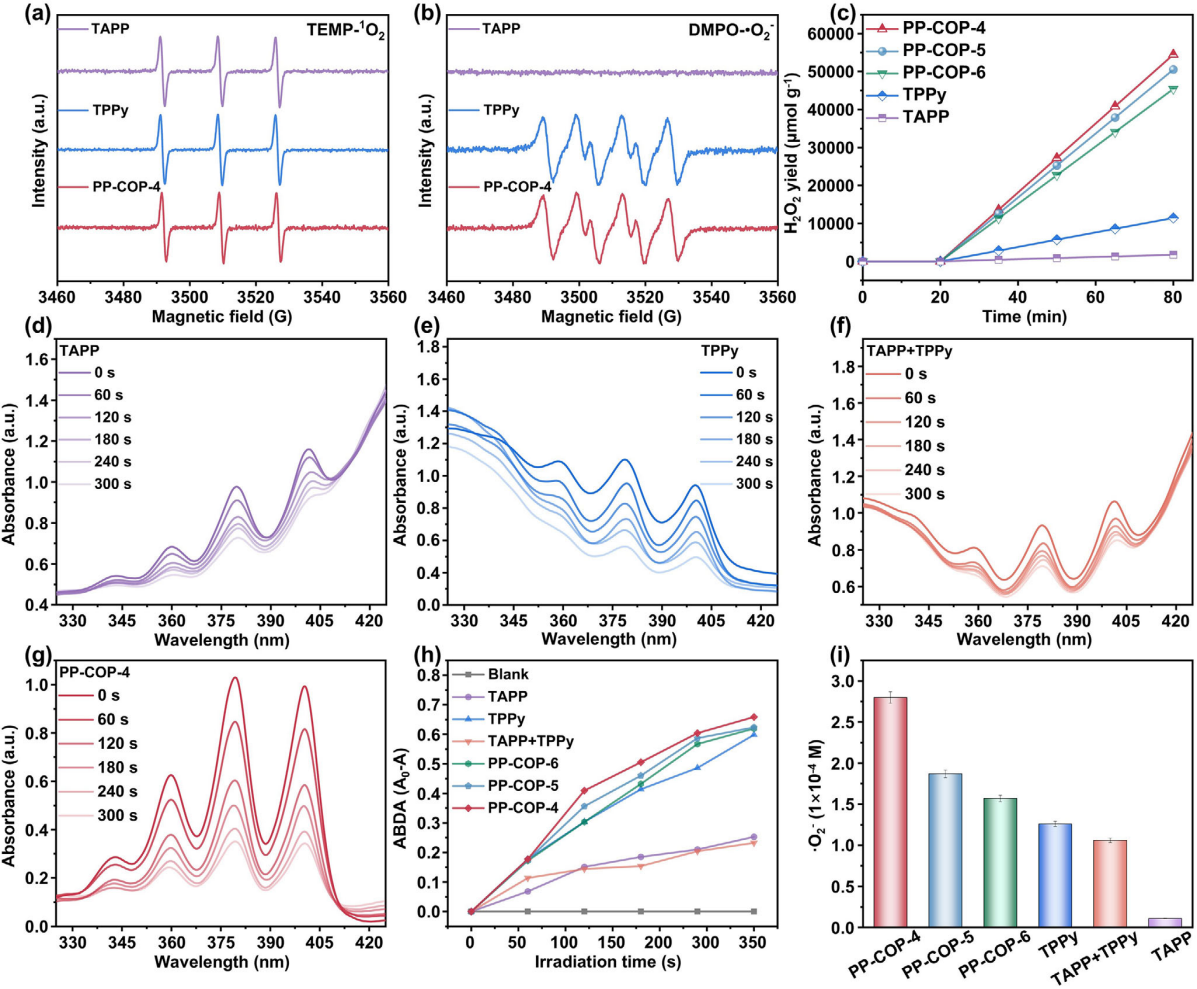
a) The ESR signals of ^1^O_2_ captured by TEMP. b) The ESR signals of •O_2_^−^ captured by DMPO. c) Photocatalytic H_2_O_2_ production performance for PP‐COPs **4**–**6**, TPPy and TAPP (Photocatalytic conditions: 3 mg catalyst, 27 mL water, 3 mL benzyl alcohol, 300 W Xe lamp, λ ≥ 365 nm). Absorption of the samples under a lamp with 365 nm: d) ABDA solution containing TAPP, e) ABDA solution containing TPPy, f) ABDA solution containing the mixture of TAPP and TPPy, and g) ABDA solution containing PP‐COP‐**4**. h) Absorption quenching percentage of ABDA solutions in the presence of PP‐COPs and its relative monomers along with the exposure time. i) NBT method for detecting the concentration of •O_2_^−^ generated by TAPP, TPPy, and PP‐COP **4–6**.

Since porphyrin and pyrrolo[3,2‐*b*]pyrrole units have been demonstrated to serve as active centers for generating ROS, specifically for ^1^O_2_ and •O_2_^−^, respectively.^[^ ^17b^ ^]^ We conducted electron spin resonance (ESR) experiments to evaluate the ROS generation capabilities of PP‐COPs. Using 2,2,6,6‐tetramethylpiperidine (TEMP) and 5,5‐dimethyl‐1‐pyrroline‐*N*‐oxide (DMPO) as scavengers for ^1^O_2_ and •O_2_^−^, we observed that ^1^O_2_ was detected in MeCN solutions of TAPP, while TPPy generated both ^1^O_2_ and •O_2_^−^ species under light illumination. Furthermore, PP‐COP‐**4** exhibited characteristic signals for both ^1^O_2_ and •O_2_^−^ (**Figures** **3**a,b), confirming its maintained ROS generation capability. This finding underscores the effectiveness of the “self‐marketing & cooperation” strategy for designing efficient photocatalysts for ROS‐mediated photocatalytic oxidation reactions.^[^ ^27^ ^]^

Due to its superior photoelectric properties and ROS generation capabilities, PP‐COPs were further conducted to evaluate its photocatalytic performance in H_2_O_2_ production. Prior to the experiment, PP‐COPs were uniformly dispersed in solution by ultrasonic treatment to facilitate the generation and transfer of H_2_O_2_. The excellent dispersibility of the PP‐COPs can be clearly demonstrated by the distinct Tyndall effect observed when their colloidal suspensions are irradiated with a laser beam (Figure S17, Supporting Information). Initially, the experiments were performed under pure water conditions without sacrificial agents and visible light irradiation (300 W Xe lamp). The quantification of H_2_O_2_ was conducted using a UV–vis spectrophotometer and the iodometric method.^[^ ^28^ ^]^ As shown in Figure S18 (Supporting Information), PP‐COP‐**4** exhibited a distinct wavelength dependence for hydrogen peroxide production, with the highest yield of 2758 µmol g^−1^ h^−1^ observed under 365 nm irradiation, compared to 420 and 550 nm. The hydrogen peroxide yield for PP‐COP‐**5** and PP‐COP‐**6** were determined to be 2507 and 2256 µmol g^−1^ h^−1^, respectively (Table S1, Supporting Information). The apparent quantum yields (AQY) at 365 nm for PP‐COPs **4–6** were calculated to be 1.75%, 1.29%, and 0.33%, respectively, and the solar‐to‐chemical energy conversion efficiencies (SCC) were determined as 0.28%, 0.22% and 0.13%, respectively (Figures S19, S20, Supporting Information).^[^ ^29^ ^]^ This finding sufficiently demonstrates that the PP‐COPs with dual photoactive sites containing porphyrin and pyrrolo[3,2‐*b*]pyrrole groups can serve as effective catalysts for the photosynthesis of hydrogen peroxide. Additionally, we found a positive correlation between the electron‐donating capacity of PP‐COPs substituents and the resulting H_2_O_2_ yield. This can be rationalized by the increased electron density at the pyrrole[3,2‐*b*]pyrrole core, which improves the charge transfer rate and promotes the kinetics of the photocatalytic ORR process.^[^ ^30^ ^]^

To obtain a higher yield of H_2_O_2_, various sacrificial agents including methanol, ethanol, isopropanol, and benzyl alcohol (BA) were introduced into the reaction system. As shown in Figure S21 (Supporting Information), when benzyl alcohol was used as a sacrificial agent, the highest yield of H_2_O_2_ was obtained. Therefore, benzyl alcohol was used as a sacrificial agent in the subsequent experiments. Upon initiation of light irradiation, the H_2_O_2_ yields of PP‐COPs **4–6** increased linearly, reaching 54 488, 50 526, and 45 430 µmol g^−1^ h^−1^, respectively. The corresponding SCC efficiencies were determined to be 0.89%, 0.80%, and 0.73% (Figure S22, Supporting Information). Notably, these yields have surpassed most reported values for metal‐free COF/COP‐based photocatalysts.^[^ ^7^, ^31^ ^]^ Additionally, to evaluate the contribution of the COPs to catalysis, we examined the activities of TAPP and TPPy. The results showed the yields of 1746 and 11 456 µmol g^−1^ h^−1^ for TAPP and TPPy, respectively. This finding suggested that the expanded conjugated structures of PP‐COPs can effectively promote H_2_O_2_ generation, which can be attributed to the cumulative and synergistic effect of two separated oxygen reduction sites.

To verify this assumption, we conducted quantitative comparative experiments to evaluate the ROS generation capabilities of various systems. 9,10‐anthracenediylbis‐(methylene)dimalonic acid (ABDA) and nitro blue tetrazolium (NBT) were utilized for efficient quantitative detection of ^1^O_2_ and •O_2_^−^, respectively.^[^ ^32^ ^]^ Upon exposure to 365 nm light, the addition of TPPy, TAPP, a TPPy/TAPP mixture, and PP‐COPs **4–6** to the ABDA solution resulted in maximum quenching percentages at 5 min, with the values of 59%, 25%, 23%, 66%, 63% and 63%, respectively (Figure 3d–h; Figure S23, Supporting Information). Similarly, the NBT experiments revealed that the concentrations of •O_2_^−^ produced during the photocatalytic cycle were 1.26 × 10^−4^ M (TPPy), 0.11 × 10^−4^ M (TAPP), 1.06×10^−4^ M (TPPy/TAPP mixture), 2.8 × 10^−4^ M (PP‐COP‐**4**), 1.87 × 10^−4^ M (PP‐COP‐**5**), and 1.57 × 10^−4^ M (PP‐COP‐**6**) (Figure 3i; Figures S24–S26, Supporting Information).^[^ ^33^ ^]^ These results demonstrated the cumulative and synergistic effects of integrating both TPPy and TAPP moieties within the PP‐COPs framework, significantly enhancing ROS production efficiency.

**Figure 4:**
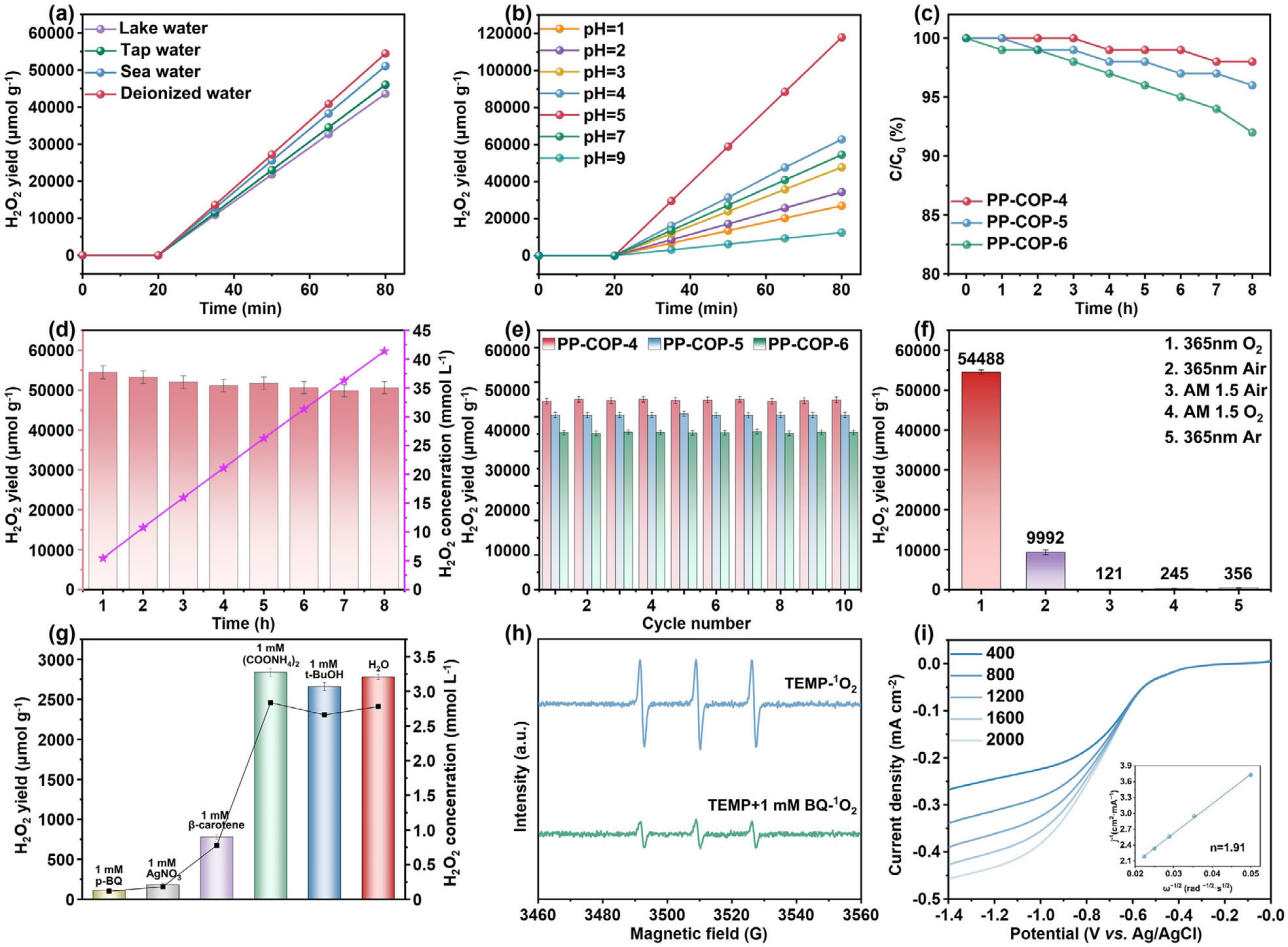
a) Photocatalytic H_2_O_2_ production performance for PP‐COP‐**4** in different water qualities. b) The effects of initial solution pH on H_2_O_2_ yield. c) Photocatalytic H_2_O_2_ decomposition experiments in water with an initial H_2_O_2_ concentration of 1 mm. d) Concentration and yield of H_2_O_2_ produced by PP‐COP‐**4** over 8 h of continuous reaction. e) Recycle results for PP‐COPs **4–6** catalyzed H_2_O_2_ production. f) Photocatalytic H_2_O_2_ production of PP‐COP‐**4** under various reaction conditions. g) ESR signals of ^1^O_2_ captured by TEMP and TEMP + 1 mM BQ. h) H_2_O_2_ yields over PP‐COP‐**4** in different photoactive species scavengers. i) Linear‐sweep RDE voltammograms of PP‐COP‐**4** at various rotating speeds.

We also carried out H_2_O_2_ generation experiments under various conditions. A linear increase in H_2_O_2_ production was observed in seawater, lake water, and tap water, with yields of 51 114, 46 057, and 43 621 µmol g^−1^ h^−1^, respectively, when PP‐COP‐**4** was used as the catalyst (**Figure** **4**a). These results demonstrate that PP‐COPs maintain high efficiency in H_2_O_2_ production across different natural water sources. However, we found that the yields of H_2_O_2_ in seawater, lake water, and tap water are lower than that in deionized water, this may be attributed to the interference from ionic components, minerals, and microorganisms present in these waters.^[^ ^34^ ^]^ Furthermore, to comprehensively ascertain the performance of PP‐COP‐**4** in different pH environments, we systematically conducted a series of experiments at pH = 1, 2, 3, 4, 5, 7, and 9. As shown in Figure 4b, we identified a discernible activity gradient, with pH = 5 > 4 >7 > 3 > 2 > 1 > 9. This result underscores the pivotal role of protonation‐induced ionic species in augmenting the photocatalytic efficiency of PP‐COPs, culminating in a remarkable H_2_O_2_ yield of 117 904 µmol g^−1^ h^−1^ at pH = 5.^[^ ^34c^ ^]^ The yield achieved a substantial 2.16‐fold increase compared to deionized water conditions, highlighting the exceptional catalytic capabilities of PP‐COPs in heterogeneous photocatalysis.

Additionally, photocatalytic H_2_O_2_ decomposition tests were conducted under light illumination with COPs. After 8 h of irradiation, the hydrogen peroxide concentrations for PP‐COPs **4–6** decreased to 98%, 96%, and 92% of the initial concentration, respectively (Figure 4c).^[^ ^35^ ^]^ In parallel, an 8‐h photocatalytic hydrogen peroxide production test was performed on PP‐COP‐**4** (Figure 4d), which showed a sustained linear increase in hydrogen peroxide concentration, maintaining a yield above 93% after 8 h. These results indicated that PP‐COP‐**4** has low activity for the decomposition of hydrogen peroxide under light irradiation, which is beneficial for the continuous and stable production of hydrogen peroxide via photocatalysis. Moreover, the PP‐COPs **4–6** can be recycled and reused ten times with slight yield deterioration (Figure 4e). The recovered PP‐COPs retained the intact structure, as evidenced by FTIR (Figure S28, Supporting Information) and XPS spectra (Figures S28–S31, Supporting Information). However, a low H_2_O_2_ yield was found under AM 1.5, which is attributed to the mismatch between the light excitation characteristics of the catalyst and the AM 1.5 spectrum (Figure 4f).^[^ ^34^, ^36^ ^]^

To gain insights into the reaction mechanism, a series of control experiments, ESR experiments, electrochemical measurements, and in situ characterizations were conducted. First, we found that the H_2_O_2_ generation was inhibited in Ar or air, suggesting O_2_ is essential for the reaction (Figure 4f; Table S2, Supporting Information). Second, to further delineate the contribution of each ROS, *p*‐BQ, *β*‐carotene, and *t*‐BuOH were employed as scavengers to probe the reaction mechanism, targeting •O_2_^−^, ^1^O_2_ and •OH, respectively (Figure 4g).^[^ ^37^ ^]^ Upon the addition of *p*‐BQ and *β*‐carotene to the reaction, the H_2_O_2_ yields were markedly reduced by 95.8% and 72.8%, respectively, compared to that in pure water, underscoring the pivotal roles of •O_2_^−^ and ^1^O_2_ in the photocatalytic production of H_2_O_2_. In contrast, the introduction of *t*‐BuOH resulted in only a 5.3% decrease in H_2_O_2_ yield, suggesting a negligible influence of •OH on the photocatalytic synthesis of hydrogen peroxide. Third, the addition of 1 m *p*‐benzoquinone (*p*‐BQ) to a mixture of COP‐**4** and water resulted in a noticeable attenuation of the ^1^O_2_ signal (Figure 4h), providing the evidence that a portion of the ^1^O_2_ is derived from the transformation of •O_2_^−^, which is consistent with previously reported mechanisms in similar systems.^[^ ^14^, ^38^ ^]^ Additionally, the incorporation of ammonium oxalate, which inhibits hole‐electron recombination, led to a 1.1% increase in H_2_O_2_ production. Notably, the presence of AgNO_3_ caused a sharp decline in H_2_O_2_ yield. Finally, Figure 4i depicts the rotating disk electrode (RDE) test results at different rotating speeds, unveiling the average electron transfer numbers (n) of 1.91 for ORR on PP‐COP‐**4**. These results demonstrate that the photocatalytic synthesis of H_2_O_2_ by PP‐COPs primarily occurs via a two‐step single‐electron transfer pathway.^[^ ^39^ ^]^ Additionally, contact angle measurements on PP‐COPs **4–6** with 1%, 2%, and 3% H_2_O_2_ concentrations (Figure S32, Supporting Information) show that the contact angle increases with higher H_2_O_2_ concentration, indicating effective H_2_O_2_ desorption. These contact angle changes reveal PP‐COPs **4–6** dynamic catalytic behavior, where surface hydrophilicity oscillates between H_2_O adsorption and H_2_O_2_ desorption phases during turnover.

**Figure 5:**
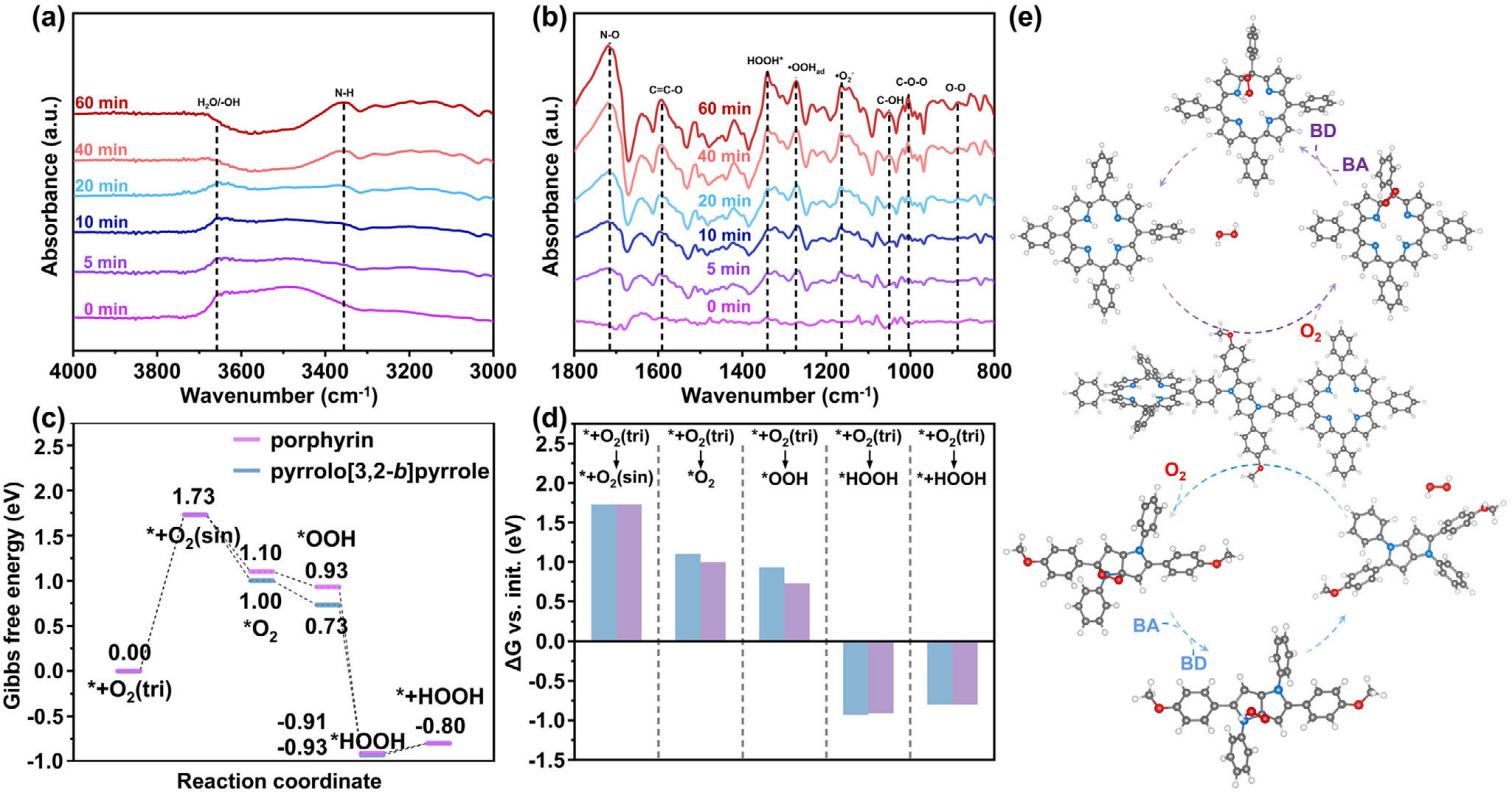
a,b) In situ FTIR spectra of PP‐COP‐**4** during the photocatalytic H_2_O_2_ evolution process. c) The Gibbs free energy diagrams for the reduction of O_2_ to H_2_O_2_ on porphyrin (purple) and pyrrolo[3,2‐*b*]pyrrole (blue). d) Gibbs free energy changes (ΔG) associated with the initial steps in ^*^+O_2_(tri) to ^*^+O_2_(sin), ^*^+O_2_(tri) to ^*^O_2_, ^*^+O_2_(tri) to ^*^OOH, ^*^+O_2_(tri) to ^*^HOOH and ^*^+O_2_(tri) to ^*^+HOOH for porphyrin (purple) and pyrrolo[3,2‐*b*]pyrrole (blue). e) Proposed mechanism for the photosynthesis of H_2_O_2_ using PP‐COP‐**4** as a catalyst.

In situ diffuse reflectance infrared Fourier transform spectroscopy (DRIFTS) was further employed to investigate the evolution in COP structures during the photocatalytic production of H_2_O_2_ (**Figures** **5**a,b). During the visible light irradiation from 0 to 60 min, the intensity of new vibrational peaks at 1271, 1162, 1006, and 885 cm^−1^ gradually increased, corresponding to the •OOH_ad_, •O_2_^−^, C‐O‐O, and O‐O bonds, respectively, confirming the involvement of superoxide anion radicals in a two‐step single‐electron oxygen reduction reaction.^[^ ^40^ ^]^ The new peak at 1592 cm^−1^ can be assigned to the stretching vibration of the C═C─O group in the pyrrolo[3,2‐*b*]pyrrole unit.^[^ ^41^ ^]^ This observation demonstrates that the pyrrolo[3,2‐*b*]pyrrole moiety in PP‐COP‐**4** undergoes structural evolution to serve as an active site for the photocatalytic O_2_ reduction reaction. Meanwhile, the significant enhancement of the C═O vibration peak at 1717 cm^−1^ indicated the oxidation of benzyl alcohol to benzaldehyde.^[^ ^42^ ^]^ Additionally, a distinct enhancement of the peak at 3358 cm^−1^ was observed, indicating the active involvement of N─H groups within the porphyrin unit in the adsorption of oxygen.^[^ ^15^ ^]^ These results indicate that both the porphyrin and pyrrolo[3,2‐*b*]pyrrole units in PP‐COP‐**4** can simultaneously facilitate the formation of H_2_O_2_.

Density functional theory (DFT) calculations were employed to elucidate the intrinsic relationship between the electronic structure of COPs and their reaction dynamics. As shown in Figure 5c,d, the calculation results revealed that ^*^O_2_ and ^*^OOH species possess lower Gibbs free energies (ΔG) when the pyrrolo[3,2‐*b*]pyrrole moieties act as the reaction centers compared to the porphyrin moieties, suggesting that the pyrrolo[3,2‐*b*]pyrrole moieties can more effectively promote the production of ^*^O_2_ and convert it sequentially to ^*^OOH and H_2_O_2_. This is also consistent with the experimental data on H_2_O_2_ yield (Figure 3a). Integrating these insights, the photocatalytic H_2_O_2_ generation mechanism by PP‐COP‐**4** is systematically illustrated in Figure 5e. First, porphyrin and pyrrolo[3,2‐*b*]pyrrole units enable the production of photogenerated electrons to promote the conversion of O_2_ to ^1^O_2_ and •O_2_^−^. Second, these ROS react with porphyrin and pyrrolo[3,2‐*b*]pyrrole units to produce endoperoxide species. Subsequently, benzyl alcohol acted as a sacrificial agent that was oxidized by holes and provided the hydrogen source to facilitate the generation of ^*^OOH species. Finally, the catalytic cycle was closed by the formation of H_2_O_2_ and the regeneration of the catalyst.

**Figure 6:**
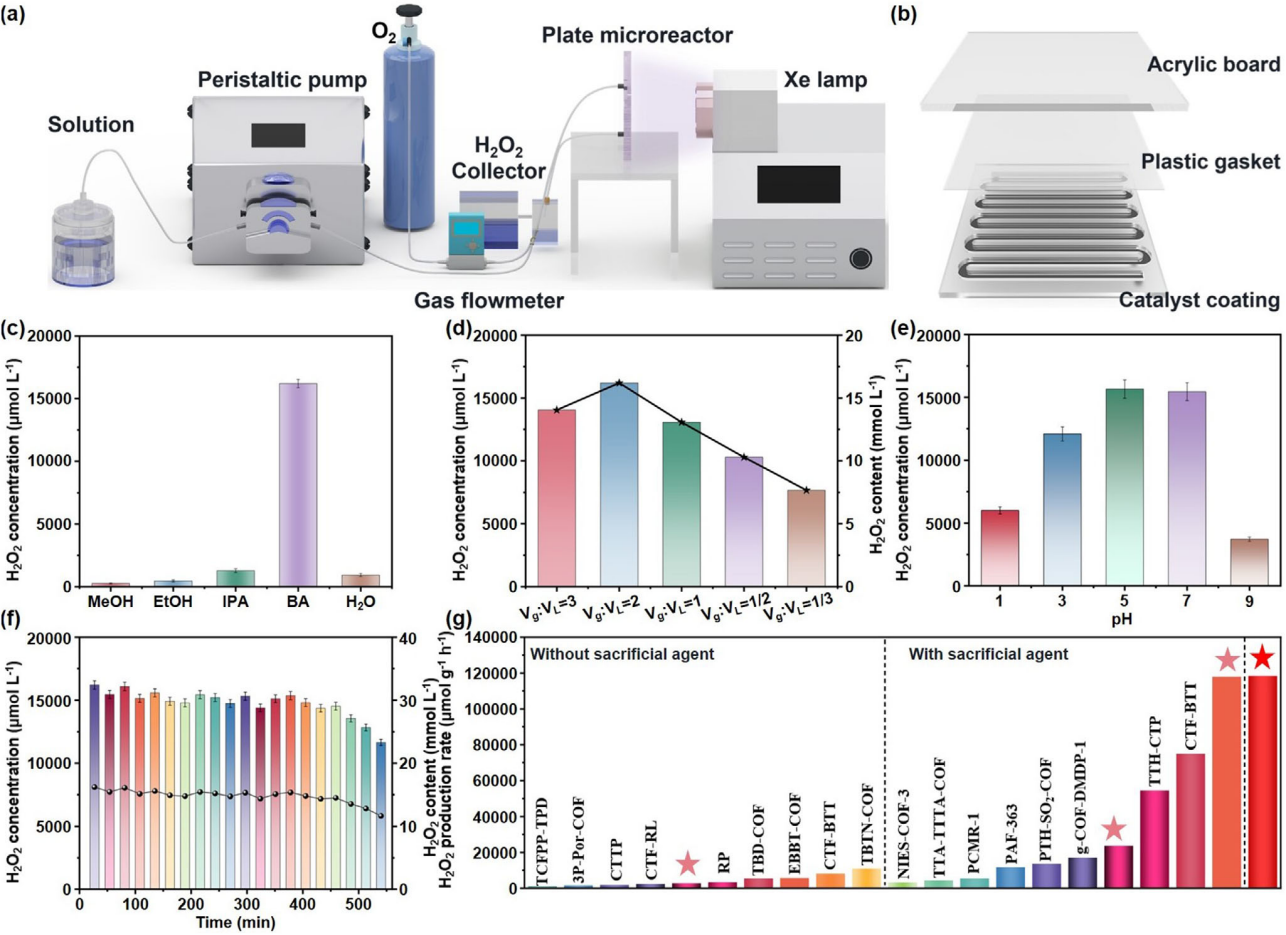
a) Schematic representation of the microreactor system and the photocatalytic H_2_O_2_ synthesis process. b) Structure diagram of the panel reactor. c) Schematic representation of H_2_O_2_ yield in different solvents. d) Schematic representation of H_2_O_2_ yield at different gas‐liquid ratios. e) Schematic representation of H_2_O_2_ yield in various pH values. f) H_2_O_2_ concentration was tracked over time in the microreactor. g) Comparison of the H_2_O_2_ yield for PP‐COP‐**4** with the reported representative metal‐free photocatalysts (The stars from left to right represent the reaction conditions of the batch reactor, pH = 7, pure water; batch reactor, pH = 7, 10% benzyl alcohol; batch reactor, pH = 5, 10% benzyl alcohol; plate reactor, pH = 7, saturated benzyl alcohol aqueous solution respectively).

To evaluate the potential of PP‐COPs for large‐scale applications, we assembled a continuous photocatalytic microplatform and immobilized PP‐COP‐**4** in a serpentine flow channel integrated into a plate reactor (**Figure** **6**a,b; Figure S33, Supporting Information). However, the use of 10% benzyl alcohol (V/V) as a sacrificial agent led to delamination issues, resulting in reactor channel clogging. To address this issue, we explored alternative alcohols, including methanol, ethanol, and isopropanol, as sacrificial agents to assess their impact on H_2_O_2_ yield. However, the H_2_O_2_ production achieved with these alcohols was consistently lower than that obtained with benzyl alcohol (Figure 6c). This difference can be attributed to the unique molecular structure of benzyl alcohol, which facilitates its oxidation by holes. Thus, a saturated aqueous solution of benzyl alcohol was employed in the following experiments to mitigate pipeline clogging caused by phase separation.

In a 10 × 10 cm area illuminated by 365 nm light, we investigated the efficiency of photocatalytic H_2_O_2_ production under different ratios of gas flow (V_g_) to liquid flow (V_L_). Initially, the spatial distribution of gas‐liquid was investigated with the V_g_/V_L_ = 0.33–3 in a total flow rate of 200 µL min^−1^, the results showed that the liquid phase within the plate microreactor is uniformly dispersed into droplets (Figure S34, Supporting Information). Subsequently, photocatalytic H_2_O_2_ production experiments were conducted under various ratios of V_g_/V_L_. The results showed an optimal H_2_O_2_ concentration of 16.2 mm within 27 min at V_g_/V_L_ = 2 (Figure 6d). This concentration is 1.95 times higher than that in the batch reactor. This is attributed to the enhanced gas‐liquid‐solid mass transfer and reduced light dissipation in solution within the microreactor.^[^ ^43^ ^]^

The effect of the pH values of the reaction solution on the yields of H_2_O_2_ in the microreactor was also explored. We found that pH = 5 was the most favorable condition, with the yield slightly higher than that in deionized water (Figure 6e). Finally, under the condition of V_g_/V_L_ = 2, pH = 7, and benzyl alcohol as the sacrificial agent, 108 mL H_2_O_2_ (14.8 mm) was continuously produced within 9 h (Figure 6f). Notably, this result has surpassed that of most polymer photocatalysts. A comprehensive comparison of photocatalytic activities among metal‐free catalysts was displayed in Figure 5g. The findings underscore that the photomicroreactor markedly augments the efficiency of H_2_O_2_ generation, thereby furnishing robust underpinnings for the scalability and industrial deployment of COPs. We further investigated the large‐scale production of H_2_O_2_ under natural sunlight. PP‐COP‐**4** demonstrated an average H_2_O_2_ production rate of 151.76 µmol g^−1^ h^−1^, accumulating 52.6 µmol L^−1^ over 4 h of continuous solar irradiation (Figure S35, Supporting Information), highlighting the significant advantages of PP‐COPs in sustainable energy conversion.

## 3 Conclusion

In summary, we have demonstrated that the rational integration of dual oxygen reduction cores into one polymer framework significantly enhances H_2_O_2_ production. By strategically employing pyrrolo[3,2‐*b*]pyrrole and porphyrin as building blocks, we successfully synthesized photoactive PP‐COPs with exceptional performance. PP‐COP‐**4** exhibited a remarkable enhancement in H_2_O_2_ yield, achieving 4.75‐fold and 31.2‐fold increases compared to the individual TPPy and TAPP units, respectively. Mechanistic investigations through DFT calculations, in situ DRIFTS, and ESR spectroscopy revealed that the two active sites operate independently yet synergistically, optimizing the ORR pathway. Furthermore, we engineered a continuous‐flow photocatalytic microreactor, which enabled scalable H_2_O_2_ synthesis with a 1.95‐fold higher concentration (16.2 mm) than conventional batch reactor. This study highlights that the precise incorporation of multiple photoactive units into one COP architecture is a highly effective strategy for designing advanced photocatalysts toward sustainable H_2_O_2_ generation. Our findings provide a foundational framework for developing multifunctional polymeric catalysts for solar‐driven chemical synthesis.

## Conflict of Interest

The authors declare no conflict of interest.

## Supporting information



Supporting Information

## Data Availability

The data that support the findings of this study are available in the supplementary material of this article.
