# Supplementary material for: Engineering Separated Dual O2 Reduction Cores into One Polymer Framework for Boosting Hydrogen Peroxide Production
Source: Adv Sci (Weinh). 2025 Jun 29;12(36):e08553. doi: 10.1002/advs.202508553 (PMC12463052; doi:10.1002/advs.202508553)
Supplement: Supplementary file 1 — Supporting Information [file ADVS-12-e08553-s001.docx]

**Supporting information**

**Engineering Separated Dual O_2_ Reduction Cores into One Polymer Framework for Boosting Hydrogen Peroxide Production**

Mengmeng Fu, Jialun He, Yingguo Li*, Jiacheng Liu, Chengsheng Wang, Jiayi Lu, Chaoyang Ma, Danfeng Jiang, Xiao Chen and Chao Yu*

*School of Environmental and Chemical Engineering, Jiangsu University of Science and Technology, Zhenjiang, Jiangsu 212003, PR China*

1. *mail:* [*liyg2022@just.edu.cn;*](mailto:liyg2022@just.edu.cn;) *chao_yu@just.edu.cn*

**Table of Contents**

Section **1**. Materials characterization

Section **2**. Synthetic procedure of Covalent Organic Polymers

Section **3**. H_2_O_2_ photosynthesis experiment

Section **4**. Figures S1-S35

Section **5**. References

**Section 1. Materials characterization**

**Materials**

All raw materials were purchased from Aladdin Corporation, and Shanghai Titan Scientific Co., Ltd. All commercial chemicals were used directly without further purification and the solvents were dried according to the standard methods.

**Characterzation**

Fourier transform infrared (FT-IR) spectra were obtained using a Bruker Tensor 37 infrared spectrometer. ^13^C cross-polarization magic angle spinning (CP/MAS) solid-state NMR spectroscopy was recorded on a 400 MHz WB solid-state NMR spectrometer. Scanning electron microscopy (SEM) images were obtained using a SU8010 electron microscope. Thermogravimetric analysis (TGA) was conducted using an SDTA851e thermo-analyzer. The X-ray analysis was performed. Photoelectron spectroscopy (XPS) was acquired using a Thermo Scientific K-Alpha spectrometer. Ultraviolet-visible diffuse reflectance absorption spectra (UV-Vis-DRS) were collected using a Shimadzu UV-2600 spectrophotometer. Electron paramagnetic resonance (EPR) spectra were obtained using a Bruker ELEXSYS II E 500 EPR spectrometer at room temperature.

**Photoelectrochemical characterization**

Photochemical tests, including electrochemical impedance spectroscopy (EIS), photocurrent response, and Mott-Schottky plots were carried out on an electrochemical workstation (CHI660C, China) in a standard three-electrode system. The synthesised samples acted as the working electrodes, the Pt mesh (1x1 cm) acted as the counter electrode, and Ag/AgCl (saturated KCl) acted as the reference electrode. A 365 nm LED lamp was employed as the light source, while a Na_2_SO_4_ (0.1 mol L^-1^) aqueous solution served as the electrolyte. The working electrodes were prepared as follows: 0.05 g photocatalyst was ground with 0.5 mL of ethanol to form a homogeneous slurry ink. Subsequently, the catalyst ink was coated onto an F-doped SnO_2_-coated glass (FTO glass, 2 x 2 cm) substrate using the doctor blade technique. All investigated electrodes exhibited a similar film thickness of approximately 10 μm.

**Panel reactor for hydrogen peroxide production**

The reactor utilized in this study is constructed from acrylic substrates, boasting an effective photolysis area of 100 cm². A catalyst dispersion was prepared by integrating 20 milligrams of COPs into a mixture consisting of 2.5 mL of deionized water, 2.5 mL of ethanol, and 200 μL of Nafion solution. Subsequent to a 30 min sonication process to ensure homogeneity, the resulting suspension was applied as a uniform film onto the substrate featuring a serpentine flow channel design. The fluid dynamics within the reactor were managed via a precision peristaltic pumping system, while the photolytic excitation was sourced from a specialized 365 nm Xe lamp. Subsequently, liquid-phase injection was carried out using a syringe pump (TYD01, Lead Fluid, China). A gas mass flow controller (GMFC-CXB, CXInstrument, China) was used to regulate the oxygen flow rate during the gas-phase injection process. The gas and liquid were mixed in a Y-configuration. The flow rate ratios of gas to liquid were selected as 3:1, 2:1, 1:1, 1:2, and 1:3, respectively, with the total flow rate set at 200 μL/min.

**Thermogravimetric Analyses (TGA)**

Thermal analysis was conducted using a SDTA851e thermogravimetric analyzer, where the samples were heated from room temperature to 800 ℃ at a rate of 1 ℃/min.

**Rotating disk electrode (RDE) measurements**

2 mg sample was dissolved in 0.5 mL ethanol and 10 μL Nafion solution under sonication for 30 minutes. The working electrode was prepared by applying a drop of the suspension (50 μL) to a disc electrode. The Ag/AgCl electrode and graphite electrode were used as the reference and counter electrodes, respectively. Linear scanning voltammetry (LSV) curves were tested in a 0.1 M Na_2_HPO_4_/NaH_2_PO_4_ solution (pH = 7) with a continuous oxygen flow at different rotational speeds. The slope of the n value according to the Koutecky-Levich (K-L) plot was calculated based on the following equation

$\text{B=0.}\text{62}\text{nFC}\text{D}^{\text{2/3}}\text{v}^{\text{-1/6}}$ (1)

$\text{j}^{\text{-1}}\text{=}\text{j}_{\text{k}}^{\text{-1}}\text{+}\text{ }\text{B}^{\text{-1}}\text{ω}^{\text{1/2}}$ (2)

In the aforementioned context, j represents the measured current density, j_k_ signifies the kinetic current density, ω is the rotating speed (rpm), F is the Faraday constant (96,485 Coulombs per mole), and C is the bulk concentration of oxygen in water (1.26 × 10^-3^ mol cm^-3^). Furthermore, ν denotes the hydrodynamic viscosity of water, which has been measured to be 0.01 cm^2^ s^-1^, while D represents the diffusion coefficient of oxygen, which is 2.7 × 10^-5^ cm^2^ s^-1^.

**The turnover frequency (TOF, h^-1^)**

$\text{TOF=}\frac{\text{Amount of }\text{H}_{\text{2}}\text{O}_{\text{2}}\text{ evolved(μmol)}}{\text{(amount of active sites in catalyst}\left( \text{μmol} \right)\text{* time}\left( \text{h} \right)\text{)}}$ (3)

**Apparent quantum yield (AQY) measurements**

The AQY values were obtained under monochromatic light irradiation at 365, 420, 550 nm. The AQY values were calculated as in the following equation:

$\text{AQY=}\frac{\text{number of photos in }\text{H}_{\text{2}}\text{O}_{\text{2}}\text{ evolution}}{\text{total number of photos}}\text{= }\frac{\text{2M}\text{N}_{\text{A}}\text{hc}}{\text{Spt}\text{γ}}$ (4)

Where M is the number of H_2_O_2_ molecules produced, N_A_ is Avogadro constant; *h* is Plank constant; c is vacuum light velocity. Here, the incident monochromatic light intensities at 365 nm, 420 nm and 550 nm were 18.3 mW/cm^2^, 19.6 mW/cm^2^ and 19.2 mW/cm^2^, respectively; the irradiated area (S) was 38.5 cm^2^; t is the irradiation time (s); λ is the monochromatic light wavelength (m).

**Solar-to-chemical energy conversion (SCC) efficiency measurements**

The SCC efficiency was determined by the photocatalytic experiments using an AM 1.5G spectrum as the light source (100 mW cm^-2^)

$\text{SCC(\%)=}\frac{\left\lfloor\text{∆G for }\text{H}_{\text{2}}\text{O}_{\text{2}}\text{ generation (J}\text{mol}^{\text{-1}}\text{)} \right\rfloor\left\lfloor\text{H}_{\text{2}}\text{O}_{\text{2}}\text{ formed (mol)} \right\rfloor}{\left\lfloor\text{total input power (W)} \right\rfloor\left\lfloor\text{reaction times (s)} \right\rfloor}$ (5)

Where ∆G is 117 kJ mol^-1^. As an example, when using PP-COP-**4** as the catalyst, the irradiated sample area was 3.14 cm^2^ during 1 hour of illumination.

**Detection of •O_2_^-^ production in solution**

The concentration of •O_2_^-^ was quantified by measuring the decay of NBT (nitro blue tetrazolium) using UV-vis spectroscopy. Before analysis, 1 mL of liquid was filtered through a 0.22 μm filter to remove any residual photocatalysts. The photocatalytic generation of •O_2_^-^ was determined by the degradation of NBT, which was detected by the absorbance change at the wavelength of 259 nm. The mole ratio of generated •O_2_^-^ and reacted NBT was 4:1.

**Detection of ^1^O_2_ production in solution**

9,10-anthracenediyl-bis(methylene)-dimalonic acid (ABDA) was used as an indicator for the detection of ^1^O_2_ in solution. A solution of 100 µg/mL of sample was dissolved in 3 mL of a solution containing 0.2 mM ABDA. The mixture was then placed in a cuvette and irradiated with a 365 nm xenon lamp. The absorption change of the sample at 378 nm was recorded using a UV-Vis spectrophotometer.

**Computational Details**

All quantum calculations were carried out by using the Gaussian 16 program.^[1]^ Geometry optimizations and the single-point energy calculations were performed at the Becke three-parameter hybrid exchange−correlation functional (B3LYP) level of theory, and 6-31+G(d) standard basis set was used for the atoms (C, H, O, N) ^[2]^. The dispersion correction schemes by Grimme (denoted as D3) were used to account for the van der Waals interactions ^[3]^. For the geometry optimization procedure, the structures were optimized until the forces were < 10^-5^ hartree/bohr and the energy change was < 10^-7^ hartree. The convergence criterion for the energy calculation during the self-consistent-field procedure was set for < 10^-8^ hartree. Solvation by water was taken into account by using the polarizable continuum model (PCM) for all system.^[4]^

The Gibbs free adsorption energy of O_2_ ($\text{∆}\text{G}_{\text{O}_{\text{2}}\text{, ads}}$) was computed according to the following equations:

$\text{∆}\text{G}_{\text{O}_{\text{2}}\text{, ads}}\text{=}\text{G}_{\text{sub+}\text{O}_{\text{2}}}\text{-}\text{G}_{\text{sub}}\text{-}\text{G}_{\text{O}_{\text{2}}}$ (6)

where $G_{{sub+O}_{2}}$ is the calculated total Gibbs free energy of the adsorption system, $G_{\mathrm{sub}}$ is the calculated Gibbs free energy of the clean substrate, and $G_{O_{2}}$ is the calculated Gibbs free energy of the O_2_ molecule.

The Gibbs free reaction energy (∆G) for each gaseous and calculated at 298.15 K, which is calculated by the binding strengths between the catalyst and the ORR intermediates.

$\text{G=E+}\text{E}_{\text{ZPE}}\text{-TS}$ (7)

Where E is the DFT calculated adsorption energy of the intermediate. Zero-point energies, thermal contributions to enthalpies, and Gibbs free energies were derived from vibrational frequency calculations with the same level of theory (B3LYP/6-31+G(d)). In the computational hydrogen electrode model, each reaction step was treated as a simultaneous transfer of the proton-electron pair as a function of the applied potential. Thus, the ΔG of ^1^O_2_ pathways were computed according to the following equations:

$\text{∆}\text{G}_{\text{*O}_{\text{2}}}\text{=}\text{G}_{\text{*O}_{\text{2}}}\text{+G}\left[ \text{2}\left( \text{H}^{\text{+}}\text{+}\text{e}^{\text{-}} \right) \right]\text{-\{G}\left( \text{*} \right)\text{+G}\left( \text{O}_{\text{2}} \right)\text{+G}\left[ \text{2}\left( \text{H}^{\text{+}}\text{+}\text{e}^{\text{-}} \right) \right]\text{\}}$ (8)

$\text{∆}\text{G}_{\text{*OO*}}\text{=G(*OO*)+G}\left[ \text{2}\left( \text{H}^{\text{+}}\text{+}\text{e}^{\text{-}} \right) \right]\text{-\{G}\left( \text{*} \right)\text{+G}\left( \text{O}_{\text{2}} \right)\text{+G}\left[ \text{2}\left( \text{H}^{\text{+}}\text{+}\text{e}^{\text{-}} \right) \right]\text{\}}$ (9)

$\text{∆}\text{G}_{\text{*OOH}}\text{=}\text{G}_{\text{*OOH}}\text{+G}\left( \text{H}^{\text{+}}\text{+}\text{e}^{\text{-}} \right)\text{-\{G}\left( \text{*} \right)\text{+G}\left( \text{O}_{\text{2}} \right)\text{+G}\left[ \text{2}\left( \text{H}^{\text{+}}\text{+}\text{e}^{\text{-}} \right) \right]\text{\}}$ (10)

$\text{∆}\text{G}_{{\text{*}\text{H}_{\text{2}}\text{O}}_{\text{2}}}\text{=}\text{G}_{{\text{*}\text{H}_{\text{2}}\text{O}}_{\text{2}}}\text{-\{G}\left( \text{*} \right)\text{+G}\left( \text{O}_{\text{2}} \right)\text{+G}\left[ \text{2}\left( \text{H}^{\text{+}}\text{+}\text{e}^{\text{-}} \right) \right]\text{\}}$ (11)

$\text{∆}\text{G}_{{\text{*}\text{+H}_{\text{2}}\text{O}}_{\text{2}}}\text{=G}\left( \text{*} \right)\text{+G}\left( {\text{H}_{\text{2}}\text{O}}_{\text{2}} \right)\text{-\{G}\left( \text{*} \right)\text{+G}\left( \text{O}_{\text{2}} \right)\text{+G}\left[ \text{2}\left( \text{H}^{\text{+}}\text{+}\text{e}^{\text{-}} \right) \right]$ (12)

$\text{G}\left( \text{H}^{\text{+}}\text{+}\text{e}^{\text{-}} \right)\text{=1/2G(}\text{H}_{\text{2}}\text{)-Eu}$ (13)

Where * is the substrate, U is the applied overpotential and e is the elementary charge. In this study, U=0 V versus reversible hydrogen electrode. *OO* represents the structure of the endoperoxide intermediate.

The methodology employed for the measurement of H_2_O_2_ concentration is in accordance with the aforementioned intermittent photocatalytic H_2_O_2_ production test method.

**Section 2. Synthetic procedure of Covalent Organic Polymers and TPPy**

**Synthesis of PP-COP-4**


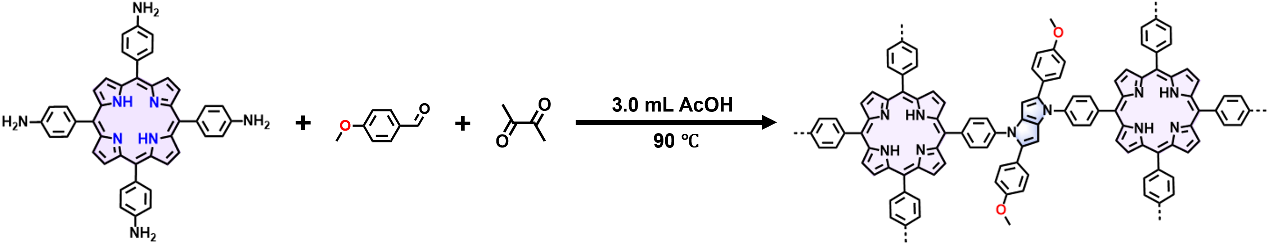


5,10,15,20-Tetrakis(4-aminophenyl)-21H,23H-porphyrin (67.5 mg, 0.1 mmol), *p*-Anisaldehyde (52.5 mg, 0.4 mmol) and *p*-Toluenesulfonic acid (7 mg) were added into 1.5 mL acetic acid within a 10 mL Pyrex tube. The mixture was subjected to sonication and heated at 90 °C for 30 minutes to conduct a preliminary reaction. After the addition of 12.9 μL of butane-2,3-dione, the reaction was allowed to proceed at 90 °C under air for 72 hours. Subsequently, the product was washed three times with acetic acid and pure water, resulting in a black precipitate. After being purified via Soxhlet extraction with THF for three days, the product was obtained as a brown powder in 71% yield (115.6 mg).

**Synthesis of PP-COP-5**


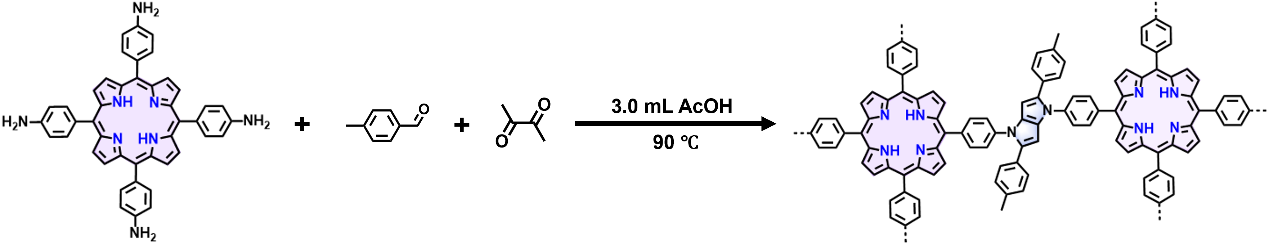


5,10,15,20-Tetrakis(4-aminophenyl)-21H,23H-porphyrin (67.5 mg, 0.1 mmol), *p*-methyl benzaldehyde (48.1 mg, 0.4 mmol) and *p*-Toluenesulfonic acid (7 mg) were added into 1.5 mL acetic acid within a 10 mL Pyrex tube. The mixture was subjected to sonication and heated at 90 °C for 30 minutes to conduct a preliminary reaction. After the addition of 12.9 μL of butane-2,3-dione, the reaction was allowed to proceed at 90 °C under air for 72 hours. Subsequently, the product was washed three times with acetic acid and pure water, resulting in a black precipitate. After being purified via Soxhlet extraction with THF for three days, the product was obtained as a brown powder in 73% yield (116.5 mg).

**Synthesis of PP-COP-6**


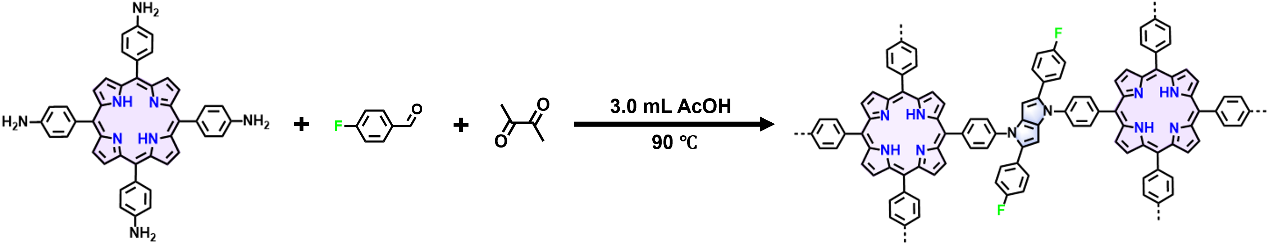


5,10,15,20-Tetrakis(4-aminophenyl)-21H,23H-porphyrin (67.5 mg, 0.1 mmol), *p*-fluorobenzaldehyde (49.6 mg, 0.4 mmol) and *p*-Toluenesulfonic acid (7 mg) were added into 1.5 mL acetic acid within a 10 mL Pyrex tube. The mixture was subjected to sonication and heated at 90 °C for 30 minutes to conduct a preliminary reaction. After the addition of 12.9 μL of butane-2,3-dione, the reaction was allowed to proceed at 90 °C under air for 72 hours. Subsequently, the product was washed three times with acetic acid and pure water, resulting in a black precipitate. After being purified via Soxhlet extraction with THF for three days, the product was obtained as a brown powder in 65% yield (103.3 mg).

**Synthesis of TPPy**

**
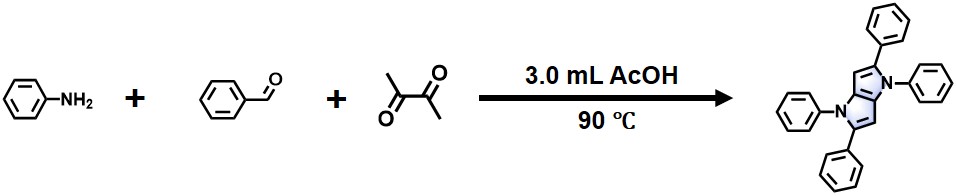
**

Aniline (18.6 mg, 0.2 mmol), benzaldehyde (21.2 mg, 0.2 mmol) and *p*-Toluenesulfonic acid (7 mg) were added into 1.5 mL acetic acid within a 10 mL Pyrex tube. The mixture was subjected to sonication and heated at 90 °C for 30 minutes to conduct a preliminary reaction. After the addition of 12.9 μL of butane-2,3-dione, the reaction was allowed to proceed at 90 °C under air for 24 hours. Subsequently, the product was washed three times each with acetic acid, pure water, and methanol, yielding a white powder with a yield of 53% (21.7 mg).

**Section 3. H_2_O_2_ photosynthesis experiment**

**Batch photocatalytic H_2_O_2_ production**

Photocatalyst (3 mg) was dispersed in a photocatalytic reactor containing 30 mL of deionized water. The catalyst was dispersed by sonication for 30 minutes, during which O_2_ was bubbled into the suspension throughout the photocatalytic process. The reaction was conducted using a 300 W Xe lamp (CEL-S500, CeauLight Co., Ltd., China) with 365 nm, 420 nm and 550 nm cut-off filters, respectively, as the light source. The concentration of H_2_O_2_ was determined using a UV-Vis spectrophotometer. During the light irradiation process, 1.0 mL of solution was removed from the photocatalytic reactor and the catalyst particles were removed using a 0.22 μm filter. The concentration of H_2_O_2_ produced was then detected by iodometric colourimetry (measured by the absorption peak at approximately 350 nm). In a typical experiment, 1 mL of the sample was filtered through a 0.22 µm Millipore filter, transferred to a 10 mL chromatographic tube, and diluted with deionized water to 5 mL. After mixing, 500 µL of C_8_H_5_KO_4_ (0.1 mol L^-1^) and 500 µL of KI (0.4 mol L^-1^) (H_2_O_2_+3I^-^+2H^+^→ I^3-^+2H_2_O) were added to the sample tube and mixed. Subsequently, the mixture was incubated in the dark for 30 minutes and analyzed by a UV-2600 spectrometer, with an absorption peak of approximately 350 nm. Throughout the entire process, the resulting H_2_O_2_ was tested on three separate occasions, with the average value being calculated. The linear relationship between the amount of H_2_O_2_ in 1 mL sample and the absorption intensity was established (see below) according to equation (4) (All samples using benzyl alcohol as the sacrificial agent were diluted 50 times for measurement).

Y=226.48A-33.25

**
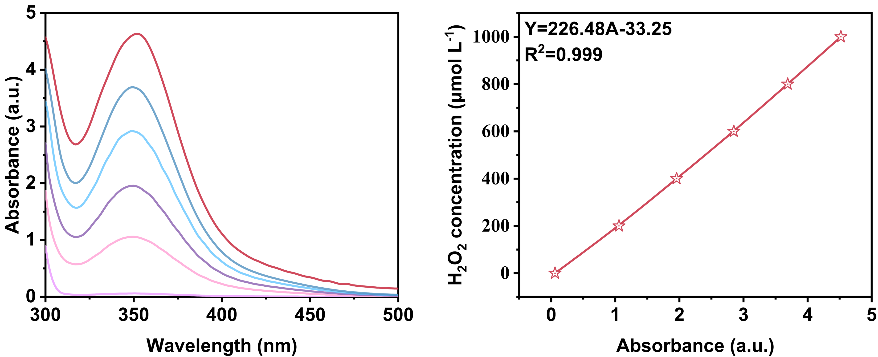
**

**Photocatalytic recycling experiment**

Photocatalytic recovery experiments were conducted using the following methods. To keep the same amount of COPs photocatalyst (3 mg) in each cycle, the COPs sample (approximately 60 mg) was suspended in 30 mL of O_2_ saturated water and irradiated for 1 hour under visible light (λ>365 nm). Collect the solid by filtration, wash three times with water and ethanol, and vacuum dry at 60 ℃ for 12 hours. Then, take 3 mg COPs for the second cycle test. The remaining solid was treated again in fresh O_2_ saturated water and irradiated for 1 hour. After collection, washing, and drying, an additional 3 mg of treated catalyst was used in the third cycle. The above procedure was repeated in the fourth and fifth cycles of testing. The testing conditions are the same as the initial experiment of photocatalytic H_2_O_2_ production.

**Section 4. Figures S1-S35**


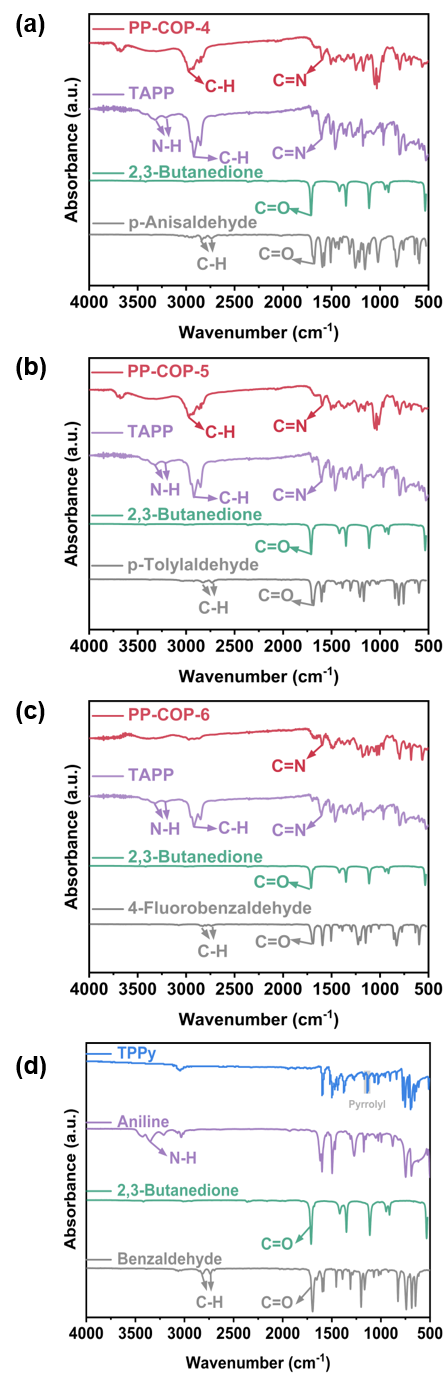


**Figure S1.** (a) FT-IR spectra of as-prepared PP-COP-**4**, TAPP, butane-2,3-dione and p-anisaldehyde. (b) FT-IR spectra of as-prepared PP-COP-**5**, TAPP, butane-2,3-dione and p-tolylaldehyde. (c) FT-IR spectra of as-prepared PP-COP-**6**, TAPP, butane-2,3-dione and 4-fluorobenzaldehyde. (d) FT-IR spectra of as-prepared TPPy, aniline, butane-2,3-dione and benzaldehyde.


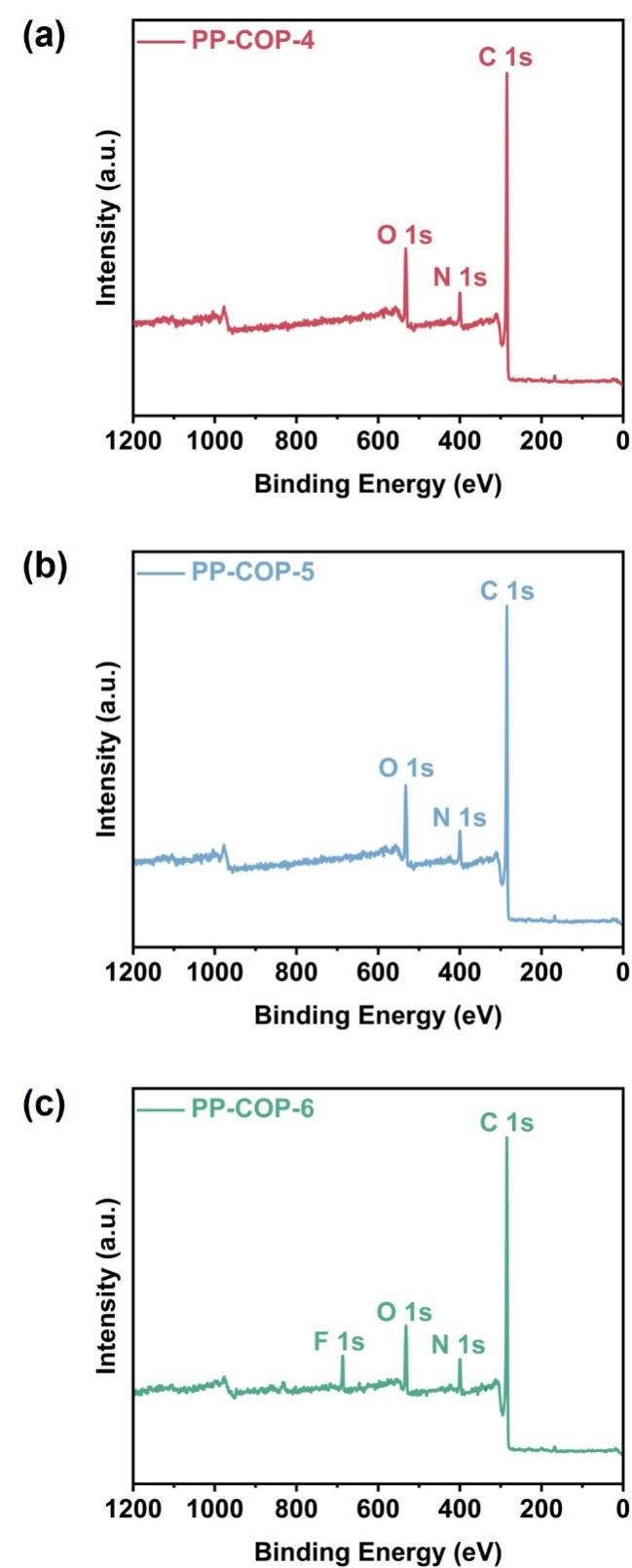


**Figure S2.** XPS characterization of (a) PP-COP-**4**, (b) PP-COP-**5**, (c) PP-COP-**6**.

**
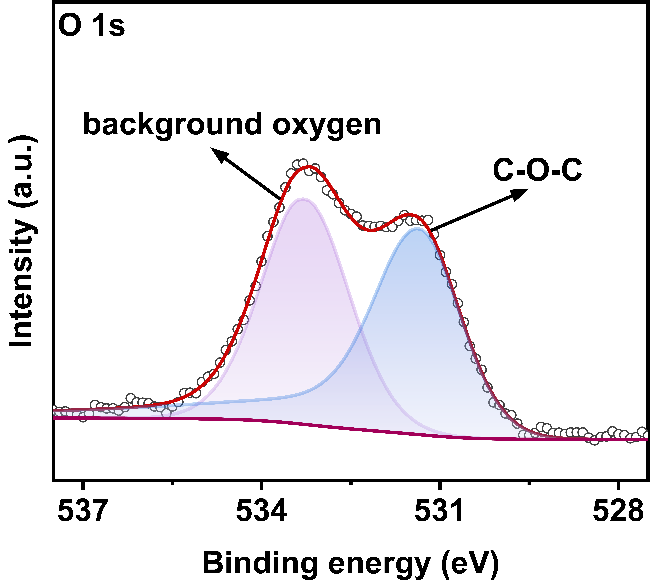
**

**Figure S3.** The O 1s XPS characterization of PP-COP-**4**.


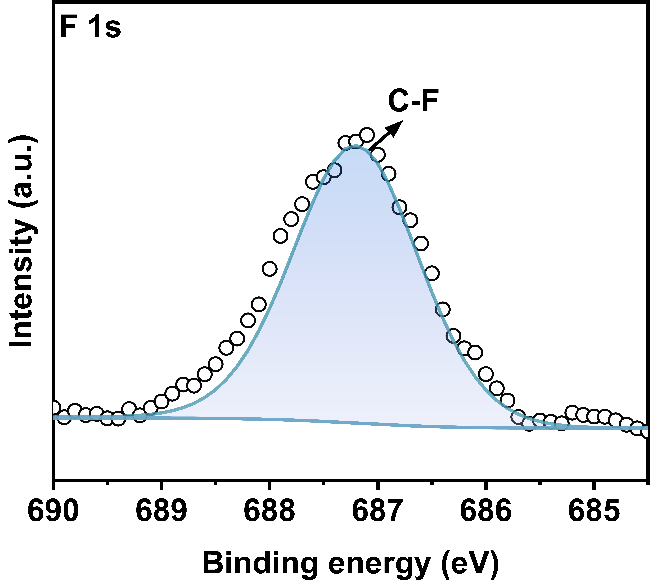


**Figure S4.** The F 1s XPS characterization of PP-COP-**6**.


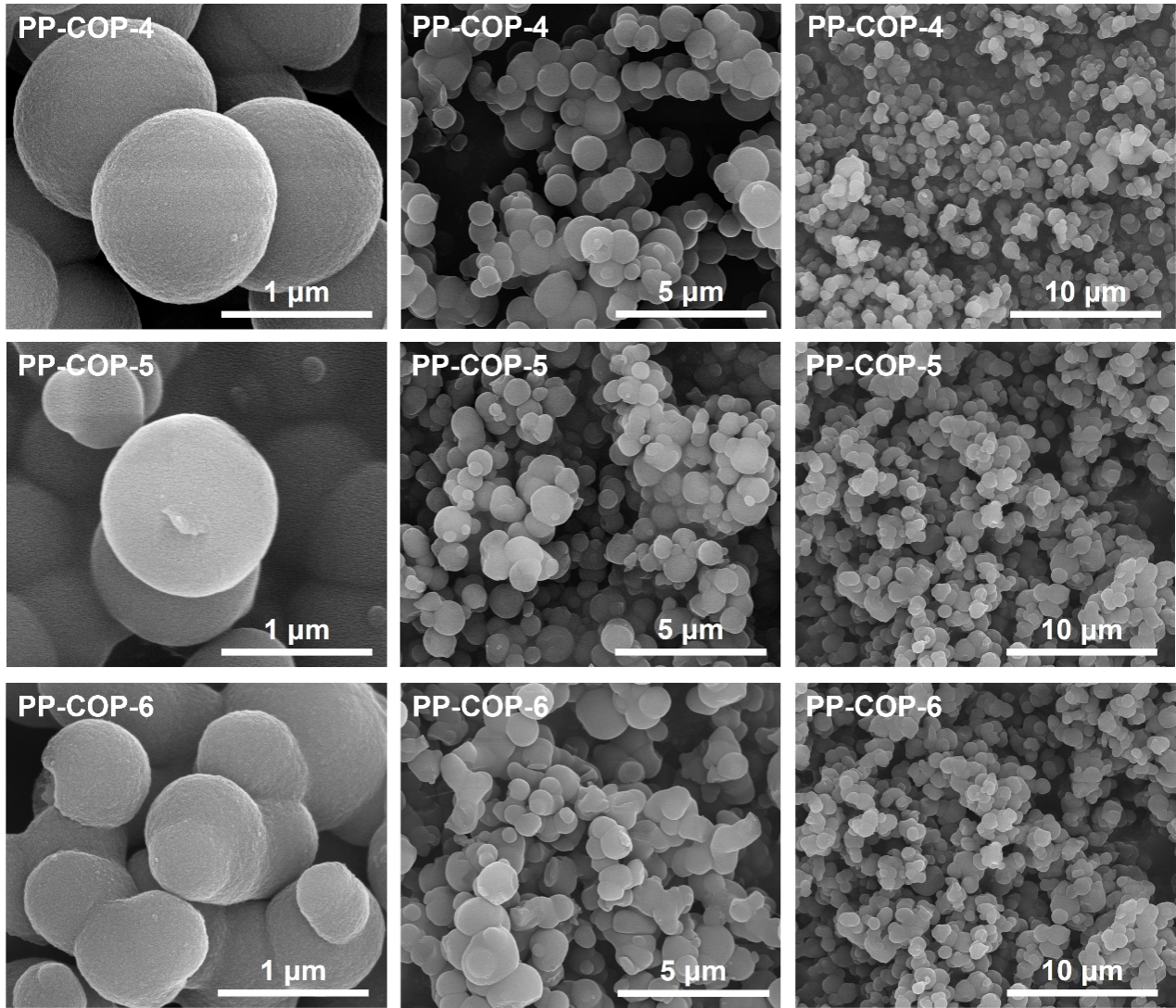


**Figure S5.** Scanning electron microscopy images (SEM) of PP-COP-**4**, PP-COP-**5** and PP-COP-**6**.


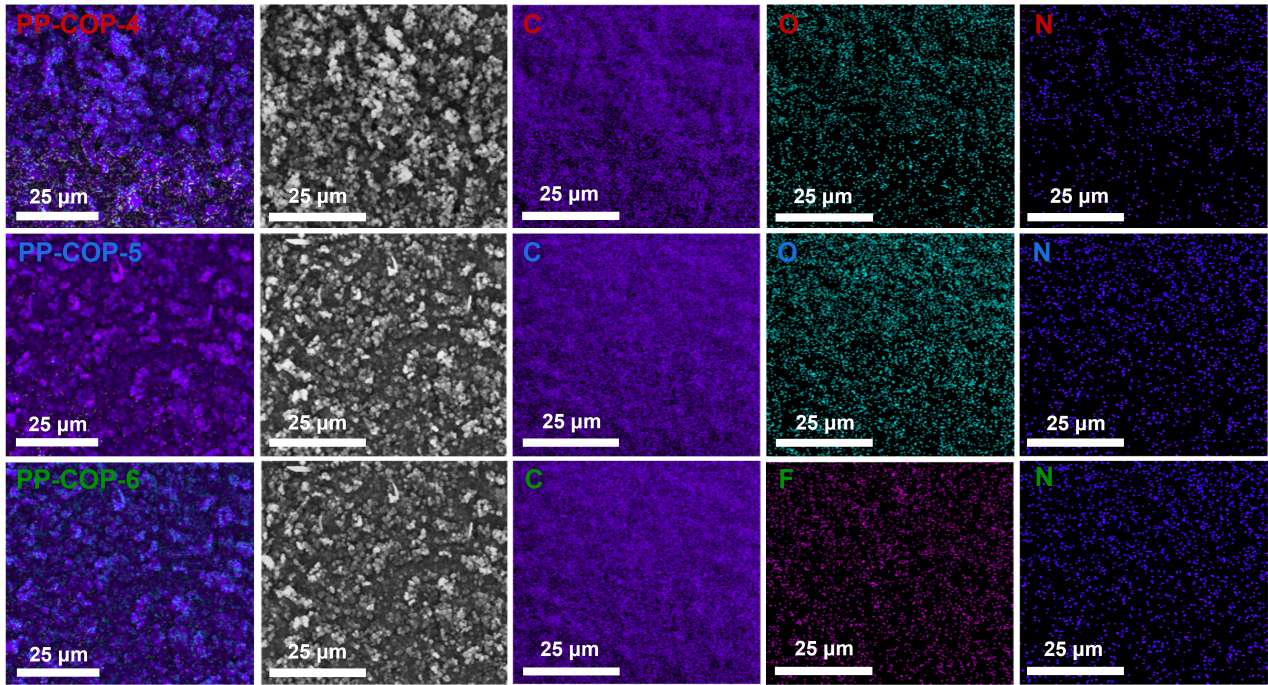


**Figure S6.** EDS mapping of PP-COP-**4**, PP-COP-**5** and PP-COP-**6**.


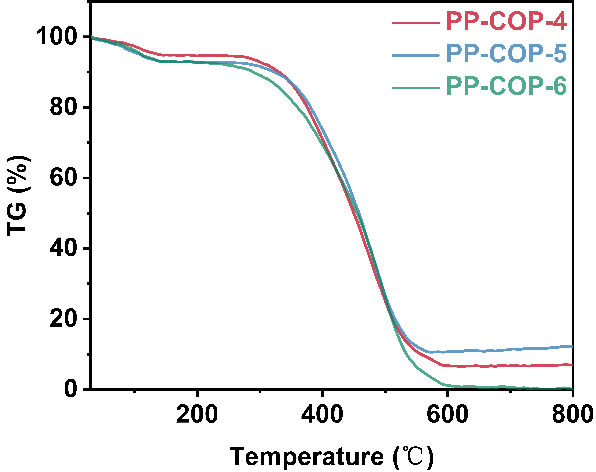


**Figure S7.** The TGA curves of PP-COPs **4-6** were measured under an oxygen atmosphere.


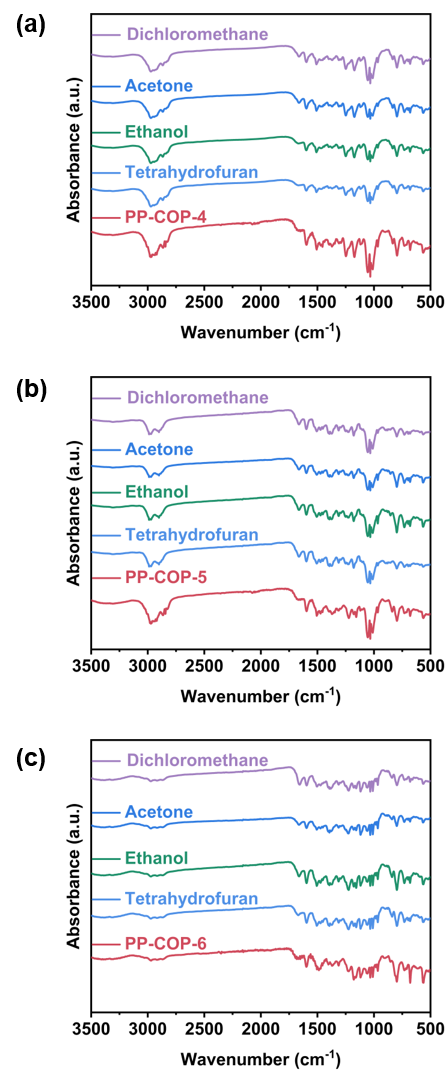


**Figure S8.** FTIR spectra of (a) PP-COP-**4**, (b) PP-COP-**5** and (c) PP-COP-**6** before and after soaking in 0.6 M solutions of dichloromethane, acetone, ethanol, and tetrahydrofuran for one week.


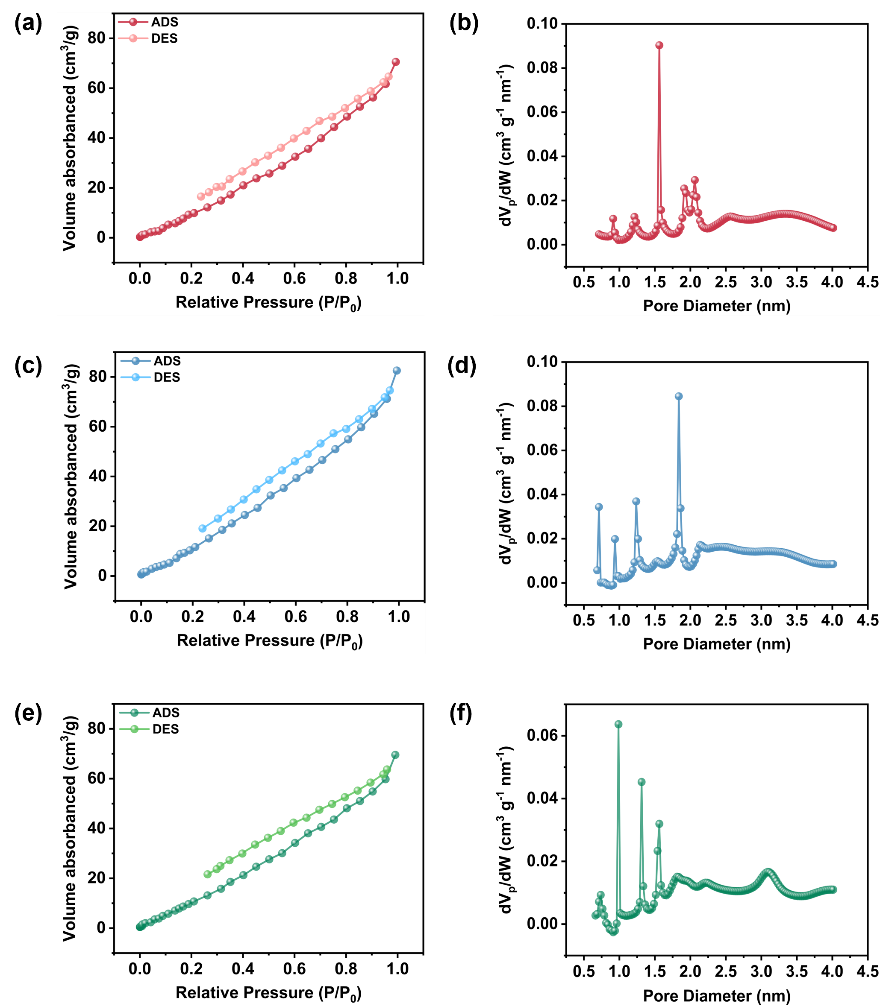


**Figure S9.** Nitrogen adsorption/desorption isotherms of (a) PP-COP-**4**, (c) PP-COP-**5** and (e) PP-COP-**6** at 77 K. (b), (d) and (f) showed the pore size of PP-COP-**4**, PP-COP-**5** and PP-COP-**6**.


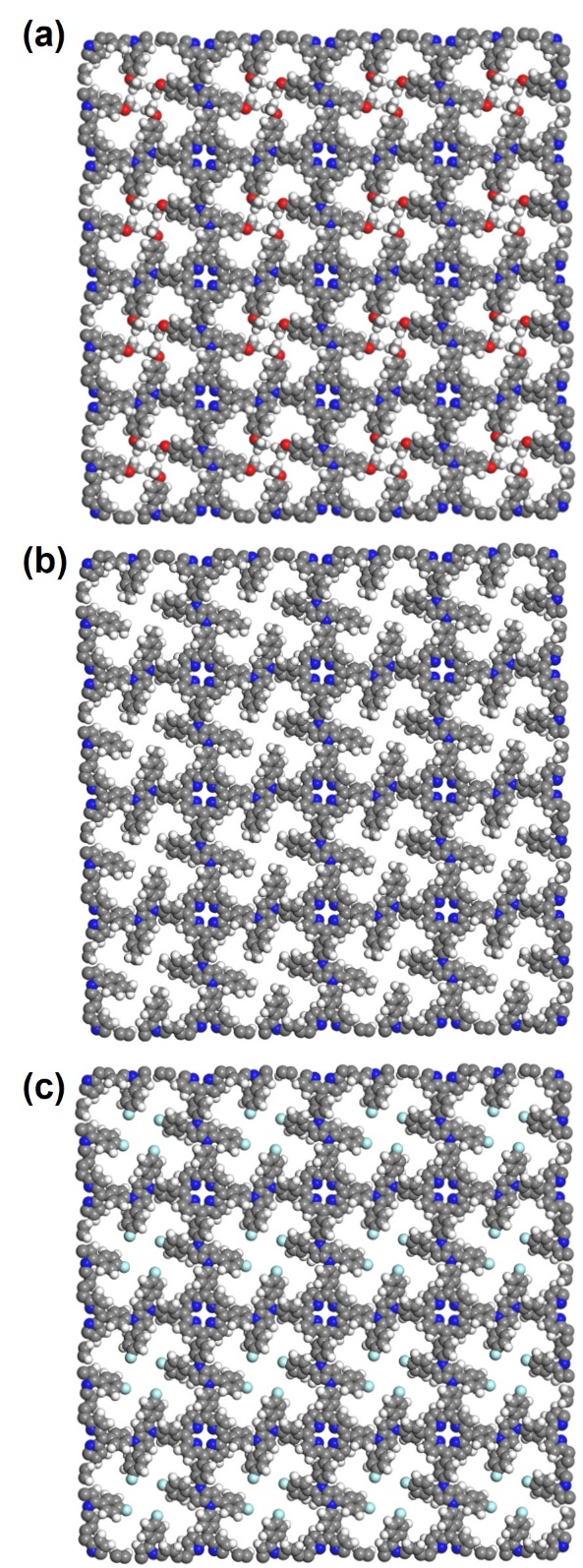


**Figure S10.** Molecular structure simulation of (a) PP-COP-**4**, (b) PP-COP-**5** and (c) PP-COP-**6**.


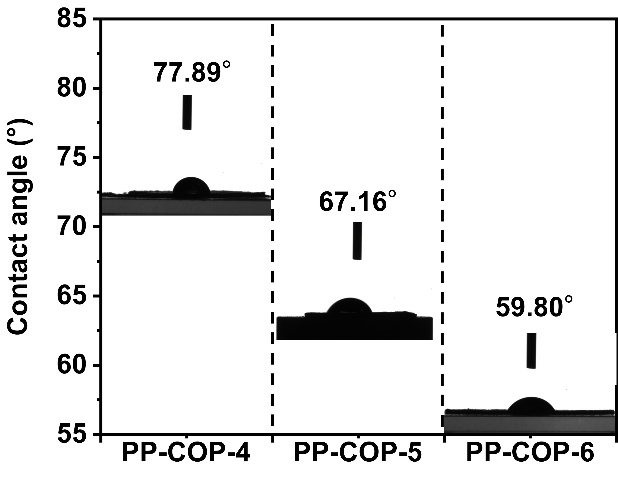


**Figure S11.** The hydrophilicity of PP-COPs **4-6** is measured via contact angle measurements.


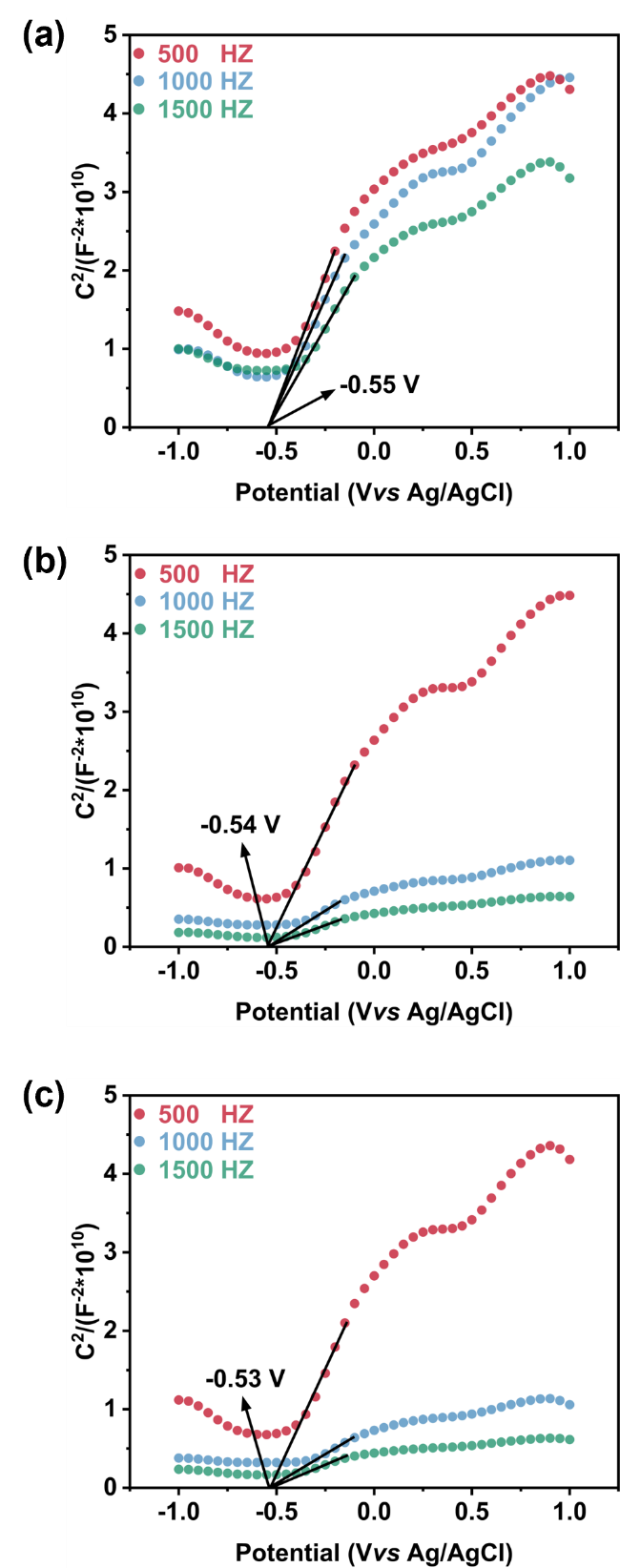


**Figure S12.** Mott-Schottky plots of (a) PP-COP-**4**, (b) PP-COP-**5** and (c) PP-COP-**6**.


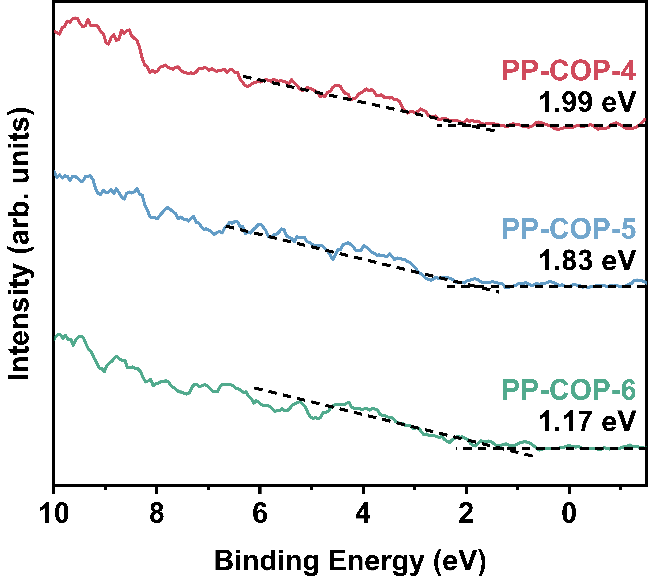


**Figure S13.** XPS valence band spectra of as-prepared PP-COPs **4-6**.


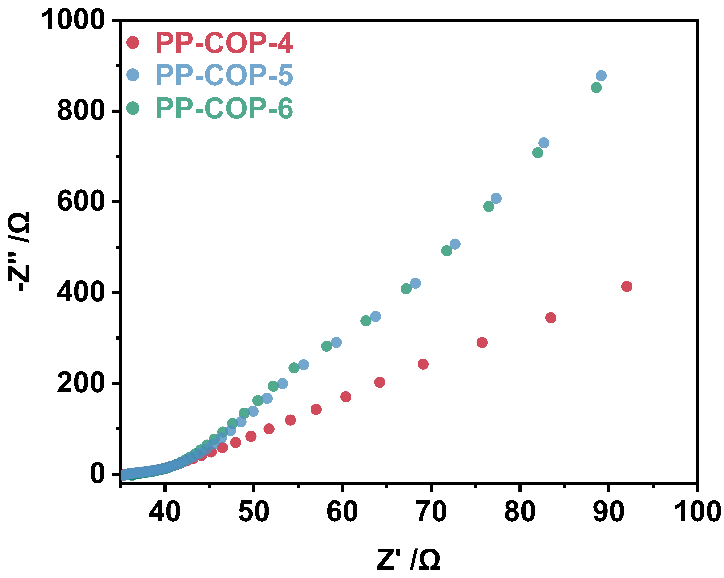


**Figure S14.** EIS characterization of PP-COPs **4-6**.


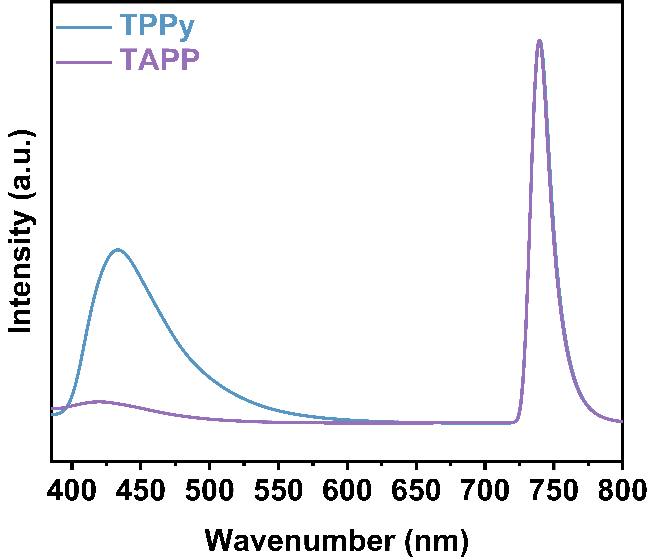


**Figure S15.** Steady-state PL spectra of TAPP and TPPy.


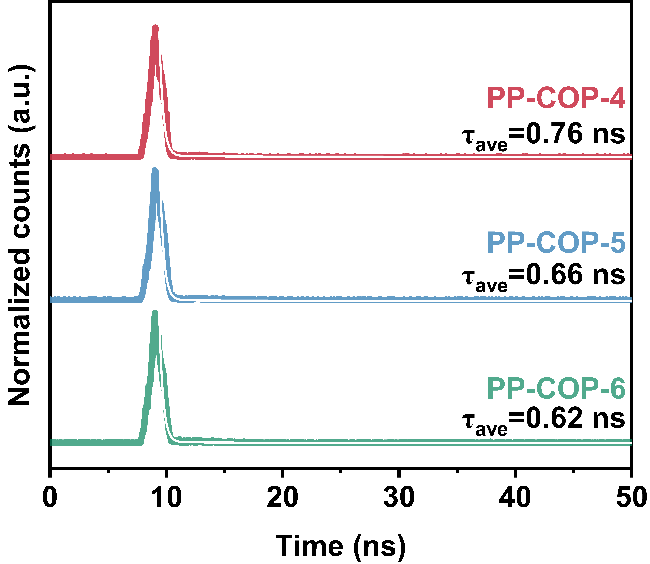


**Figure S16.** Time-resolved PL spectra of PP-COPs **4-6** upon excitation at 720 nm.


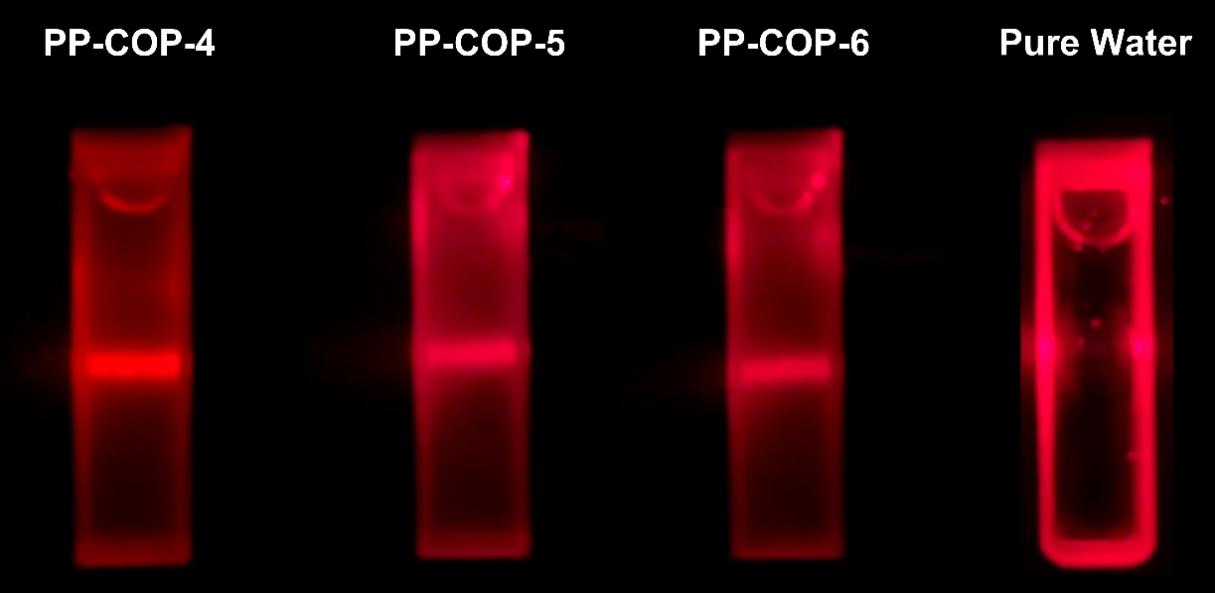


**Figure S17.** Characterization of the Tyndall effect of PP-COPs **4-6** ultrasound in deionized aqueous solutions.


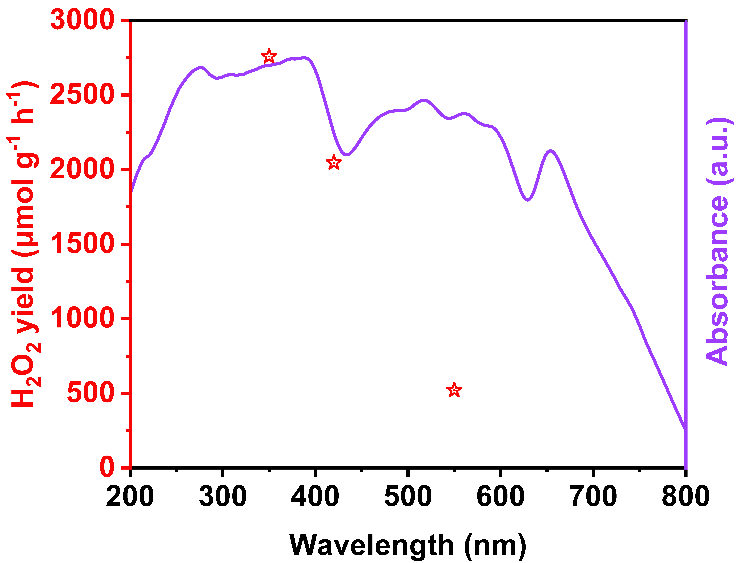


**Figure S18.** Wavelength-dependent H_2_O_2_ yield rates for PP-COP-**4** (Test conditions: 3 mg photocatalyst, 30 mL of pure water, O_2_ atmosphere, 300 W Xe lamp).


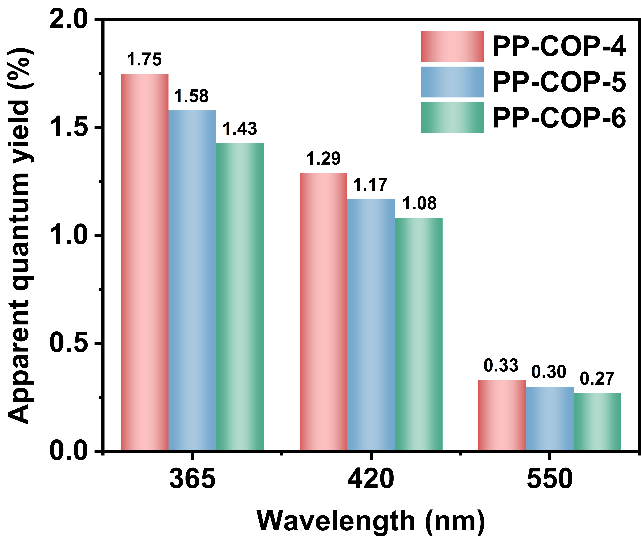


**Figure S19.** The apparent quantum yields (AQY) of PP-COPs **4-6** in H_2_O_2_ synthesis under varied light wavelengths (3 mg catalyst, 30 mL H_2_O, O_2_, 300 W Xe lamp).


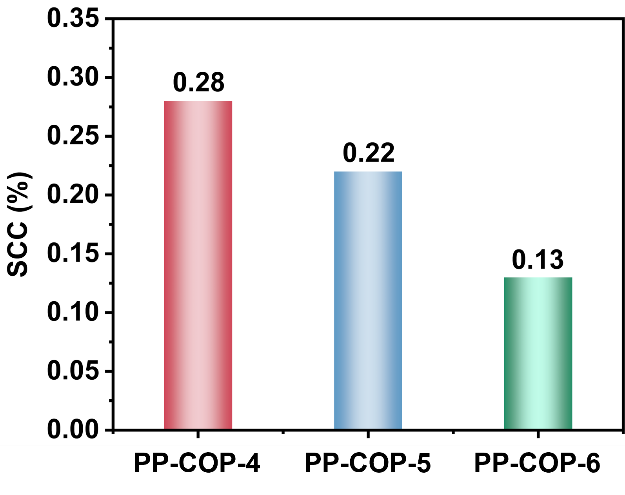


**Figure S20.** The solar-to-chemical energy conversion efficiencies (SCC) PP-COPs **4-6** in H_2_O_2_ synthesis under varied light wavelengths (3 mg catalyst, 30 mL H_2_O, O_2_, 300 W Xe lamp).


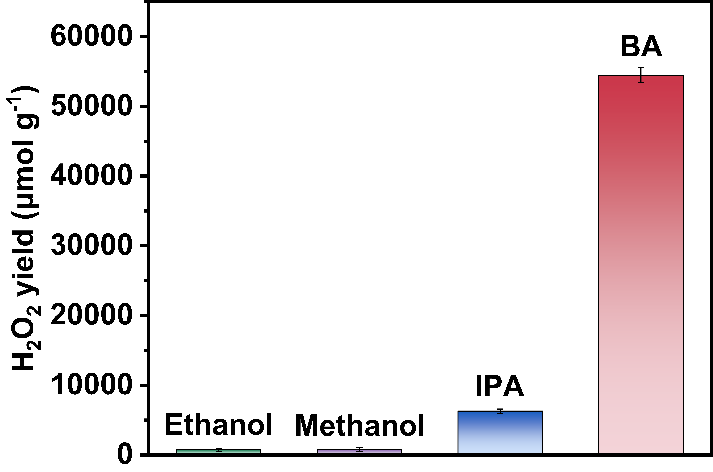


**Figure S21.** Comparison of photocatalytic H_2_O_2_ yield for PP-COPs **4-6** under different sacrificial agents at pH=7 (3 mg catalyst, 3 mL sacrificial agent and 27mL H_2_O, O_2_, 300 W Xe lamp).


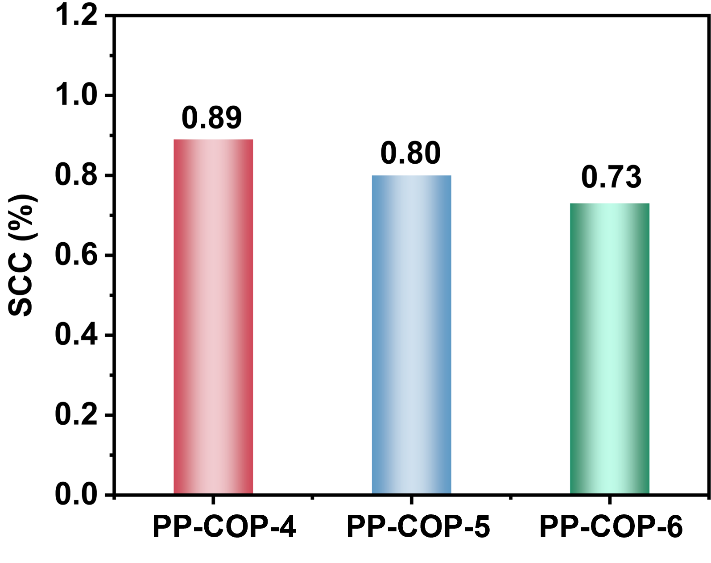


**Figure S22.** The solar-to-chemical energy conversion efficiencies (SCC) PP-COPs **4-6** in H_2_O_2_ synthesis under varied light wavelengths (3 mg catalyst, 3ml benzyl alcohol and 27mL H_2_O, O_2_, 300 W Xe lamp).


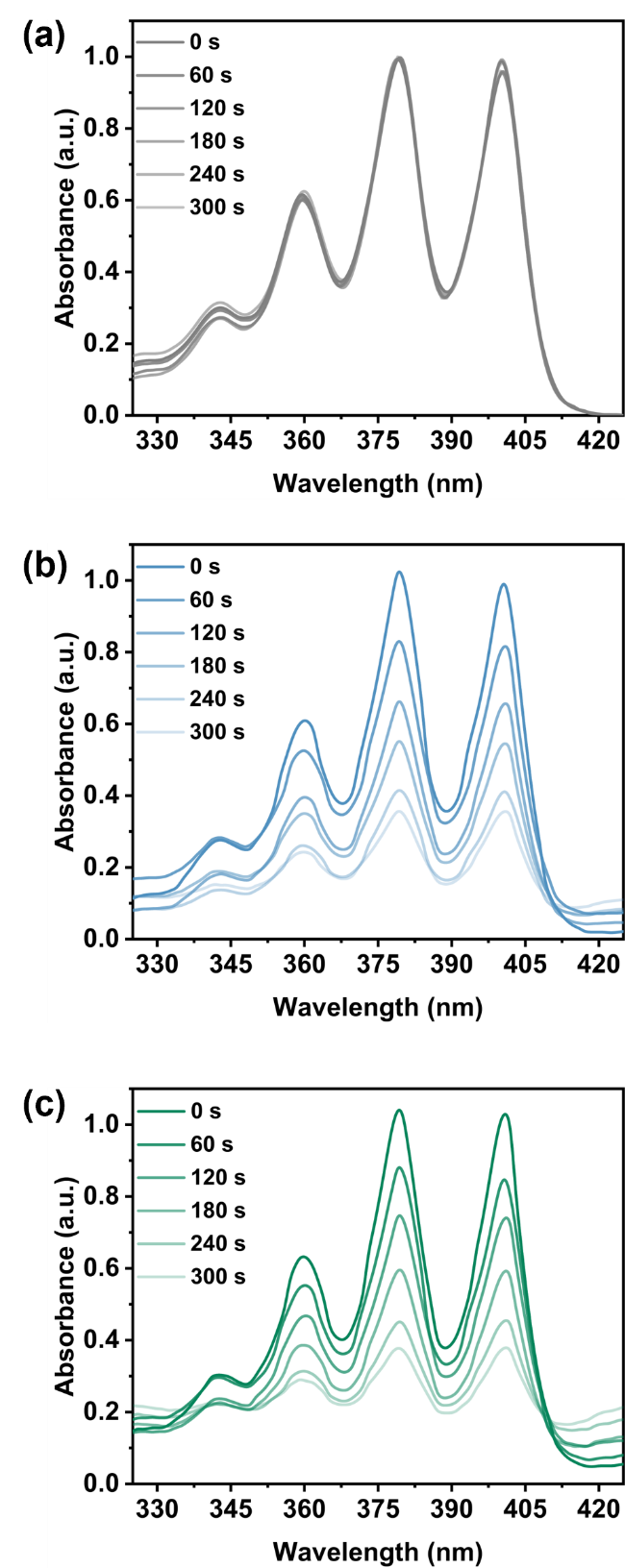


**Figure S23.** Absorption of the samples under lamp with 365 nm: (a) ABDA solution, (b) ABDA solution containing PP-COP-**5**, (c) ABDA solution containing PP-COP-**6**.


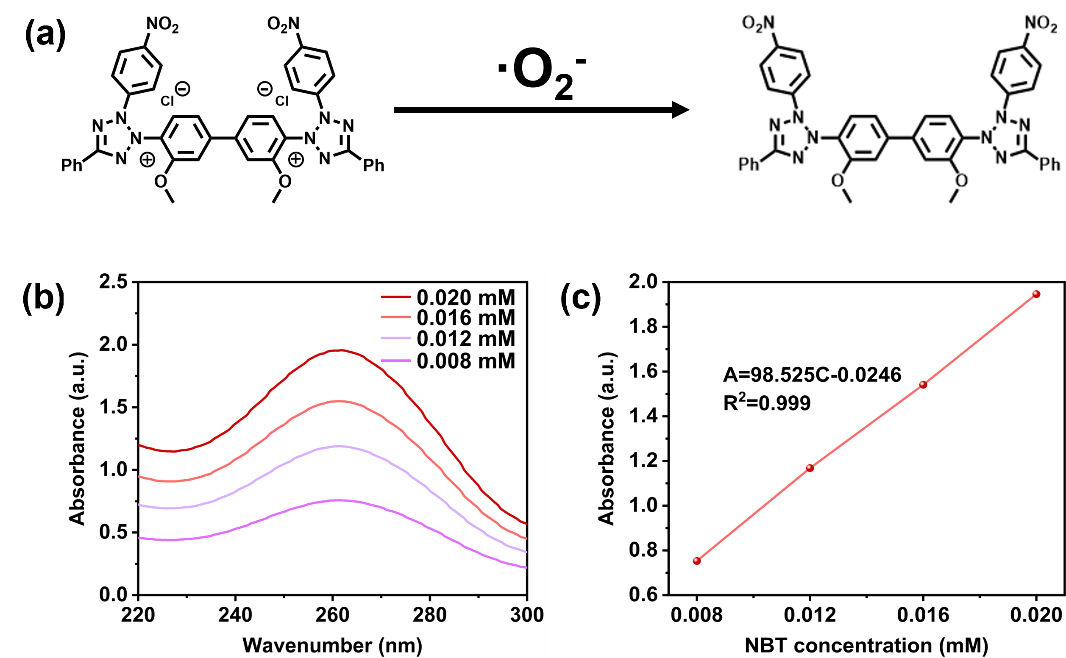


**Figure S24.** (a) Schematic diagram of NBT reaction with •O_2_^-^. (b) UV-vis characterization of different concentrations of NBT. (c) Standard curve for NBT concentration.


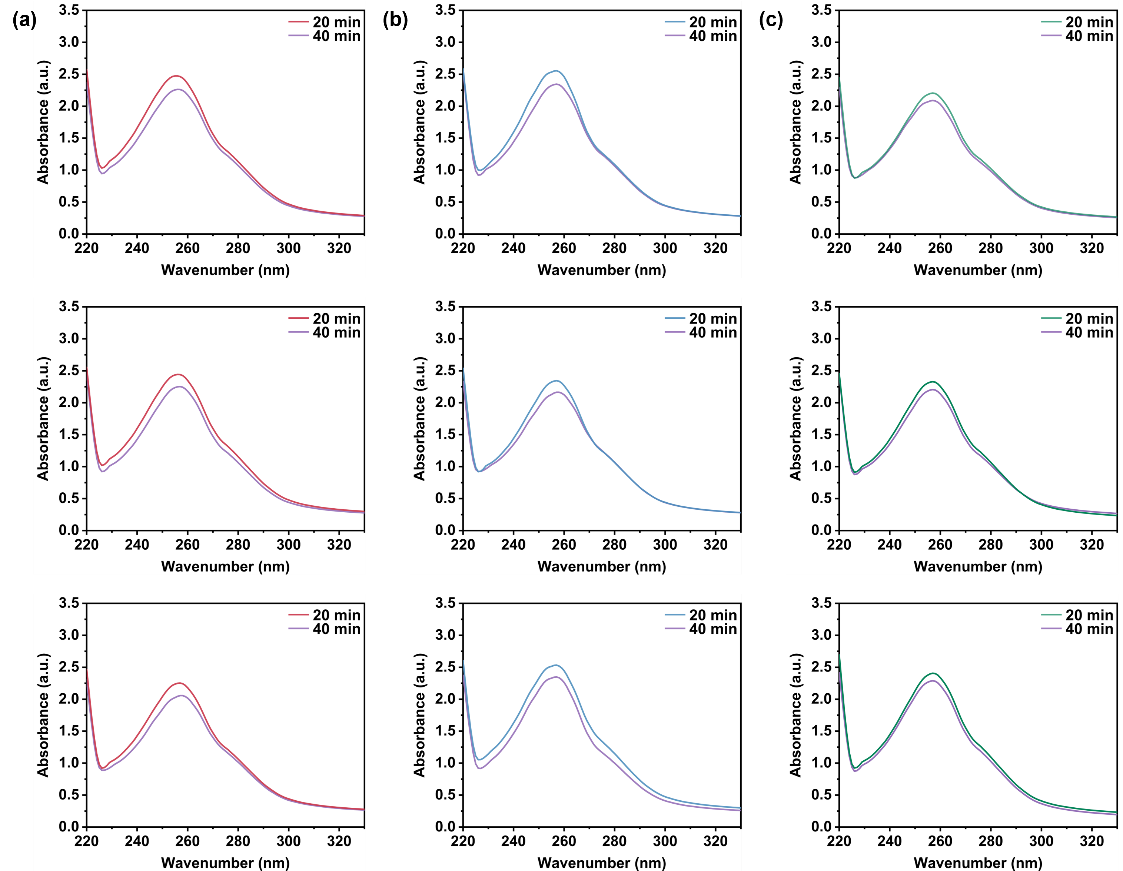


**Figure S25.** (a), (b), and (c) depict the replicate experiments using NBT for PP-COPs **4-6** respectively. Each set of experiments was repeated three times (All samples were diluted 20 times for measurement).


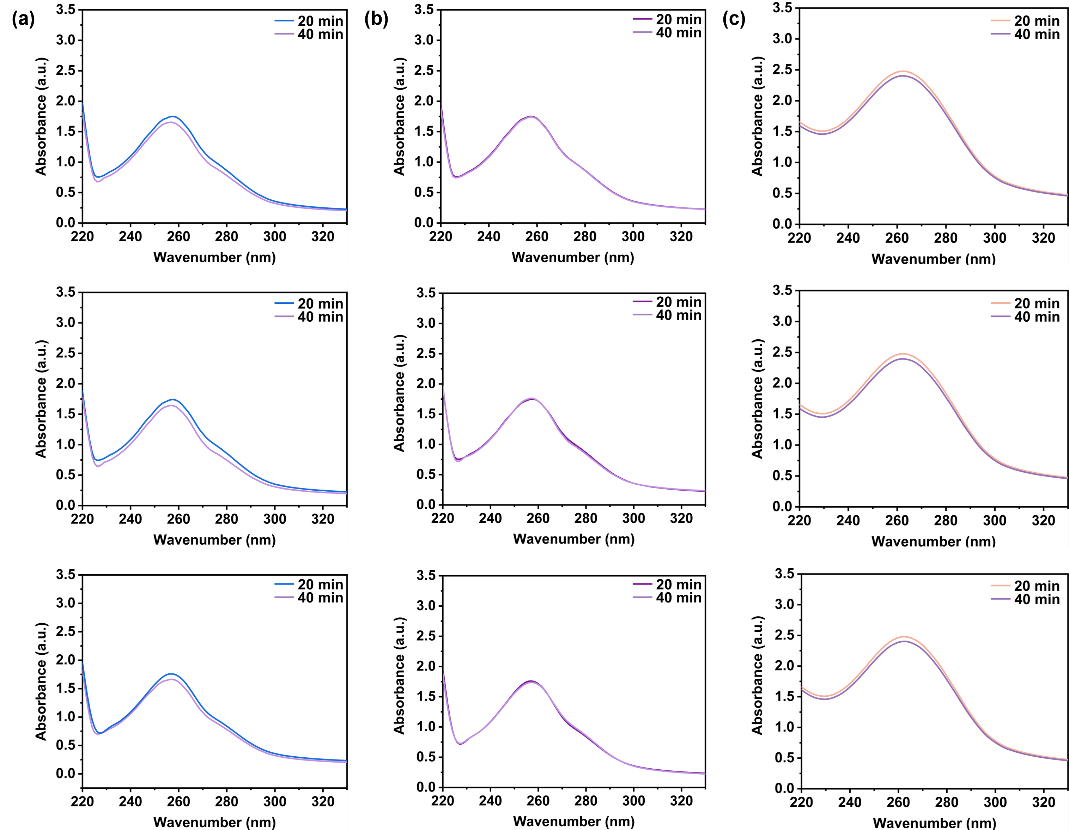


**Figure S26.** (a), (b), and (c) depict the replicate experiments using NBT for TPPy, TAPP and the mixture of TFPA and TAPP respectively. Each set of experiments was repeated three times (All samples were diluted 20 times for measurement).


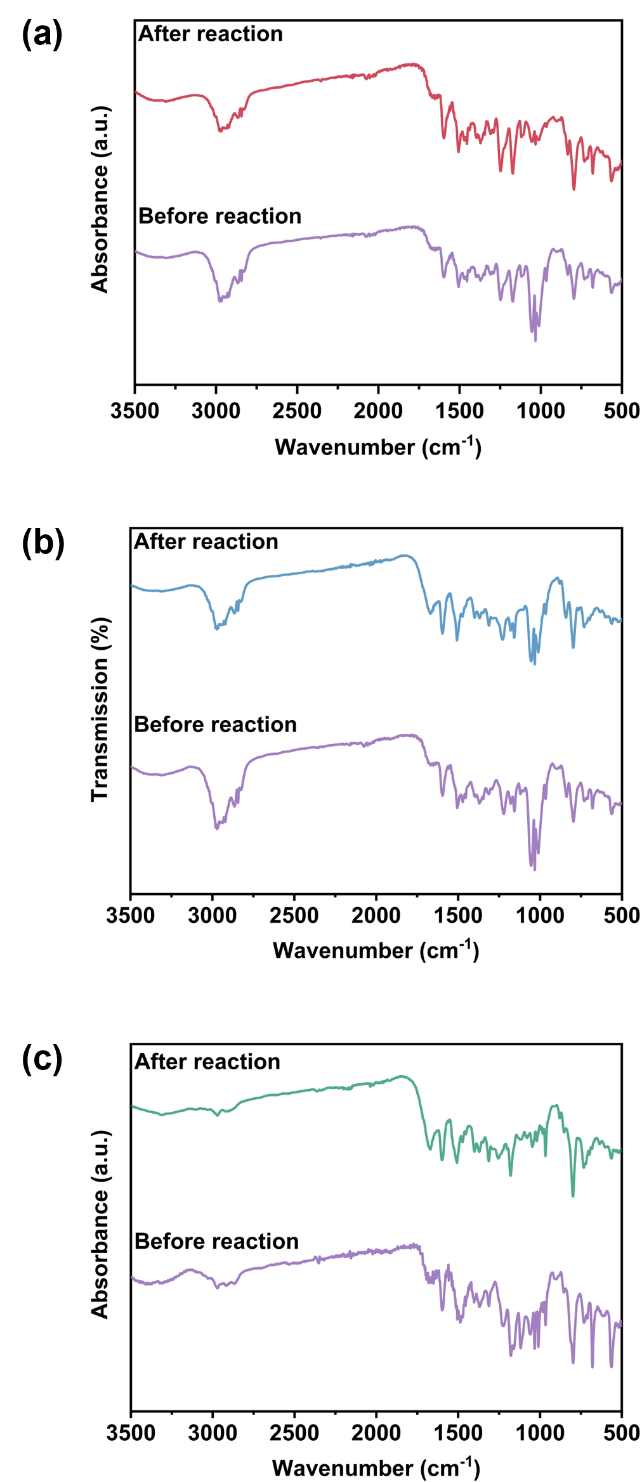


**Figure S27.** FTIR spectra of (a) PP-COP-**4**, (b) PP-COP-**5** and (c) PP-COP-**6** before and after the photocatalytic reaction.


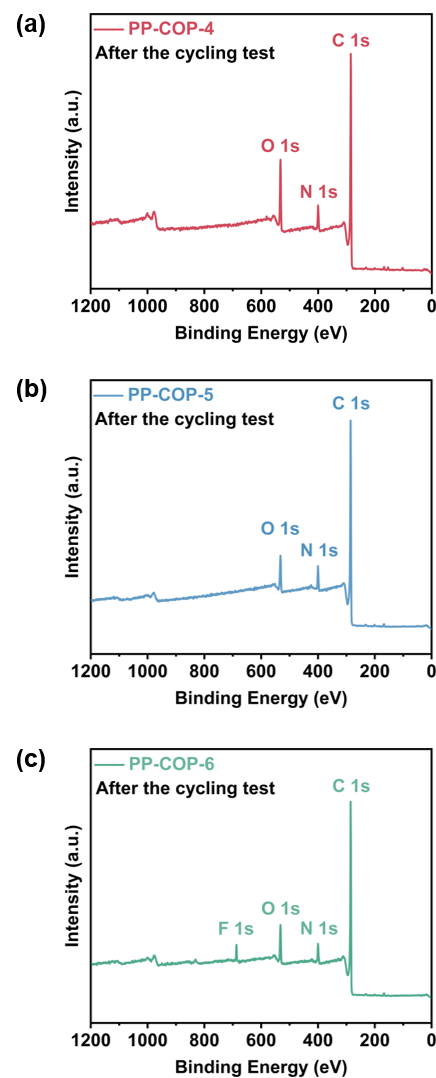


**Figure S28.** XPS characterization of (a) PP-COP-**4**, (b) PP-COP-**5**, (c) PP-COP-**6** after the photocatalytic reaction.


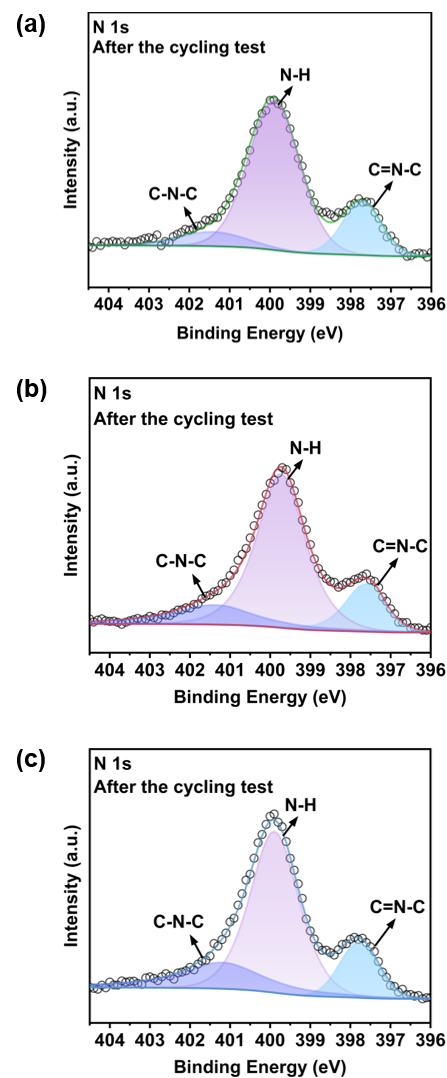


**Figure S29.** The N 1s XPS characterization of (a) PP-COP-**4**, (b) PP-COP-**5**, (c) PP-COP-**6** after the photocatalytic reaction.


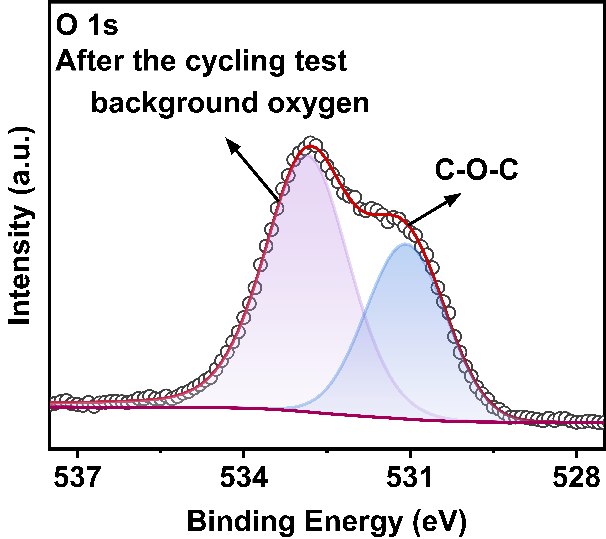


**Figure S30.** The O 1s XPS characterization of PP-COP-**4**.


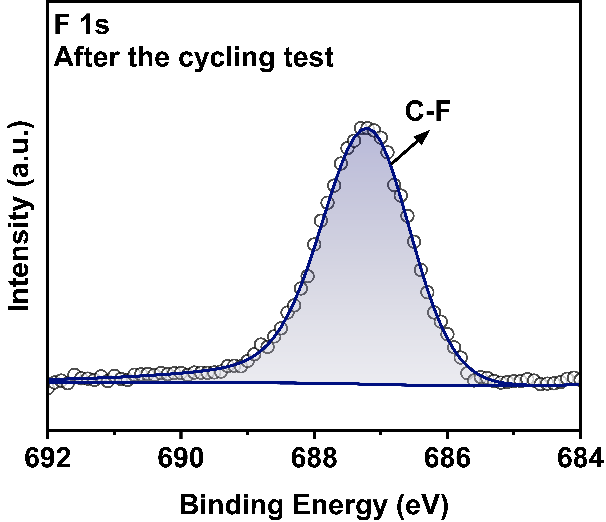


**Figure S31.** The F 1s XPS characterization of PP-COP-**6**.


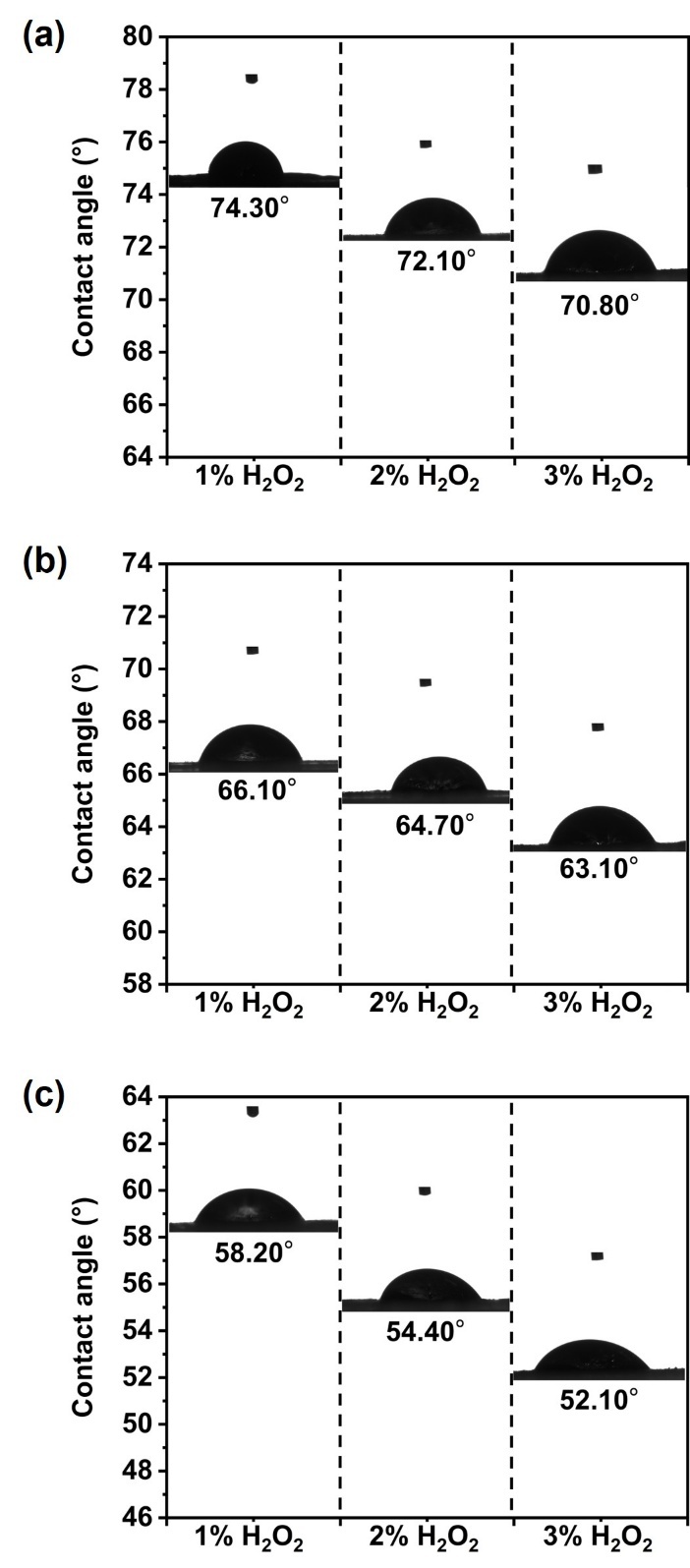


**Figure S32.** The contact angle measurements of (a) PP-COP-**4**, (b) PP-COP-**5** and (c) PP-COP-**6** in aqueous solutions of H_2_O_2_ at different concentrations.


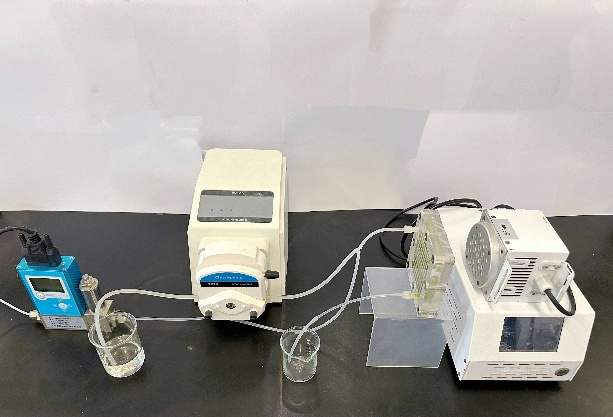


**Figure S33.** Continuous flow system.


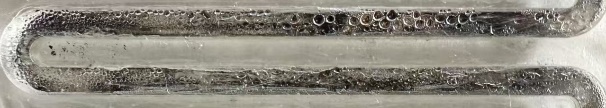


**Figure S34.** The distribution of gas and liquid in the microchannel.


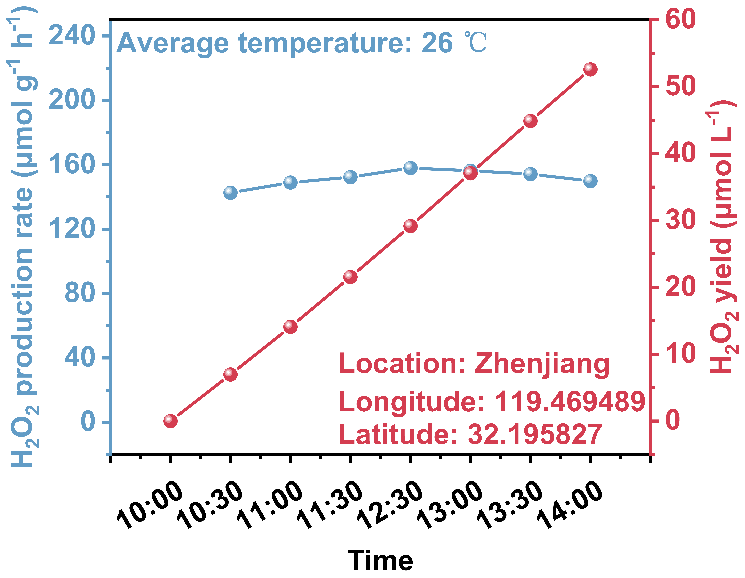


**Figure S35.** The H_2_O_2_ yield of PP-COP-**4** under sunlight.

**Table S1.** The photocatalytic yield of H_2_O_2_ generation for PP-COPs **4-6** and PP-COPs components.

| **Catalyst** | **Light source** | **Sacrificial agent** | **H_2_O_2_ production rate (μmol g^-1^ h^-1^)** |
| --- | --- | --- | --- |
| PP-COP-4 | 365nm | 10% BA | 54488 |
| PP-COP-4 | 365nm | 10% BA | 50526 |
| PP-COP-4 | 365nm | 10% BA | 45430 |
| TAPP | 365nm | 10% BA | 11456 |
| TPPy | 365nm | 10% BA | 1746 |
| PP-COP-4 | 365nm | - | 2758 |
| PP-COP-4 | 420nm | - | 2046 |
| PP-COP-4 | 550nm | - | 521 |
| PP-COP-5 | 365nm | - | 2507 |
| PP-COP-5 | 420nm | - | 1846 |
| PP-COP-5 | 550nm | - | 483 |
| PP-COP-6 | 365nm | - | 2256 |
| PP-COP-6 | 420nm | - | 1711 |
| PP-COP-6 | 550nm | - | 434 |

Reaction conditions: 3 mg catalyst, 3 mL benzyl alcohol and 27mL H_2_O were added to the photomicroreactor and continuously introduced O_2_ for 1 hour under irradiation at a

wavelength of 365 nm for reaction.

**Table S2.** Investigate the yield of H_2_O_2_ generated by photocatalysis using PP-COP-4 under different environmental conditions.

| **Catalyst** | **Atmosphere** | **Light source** | **H_2_O_2_ production rate (μmol g^-1^ h^-1^)** |
| --- | --- | --- | --- |
| PP-COP-4 | Ar | 365nm | 121 |
| PP-COP-4 | O_2_ | AM 1.5 | 245 |
| PP-COP-4 | Air | AM 1.5 | 356 |
| PP-COP-4 | Air | 365nm | 9358 |
| PP-COP-4 | O_2_ | 365nm | 54488 |

Reaction conditions: 3 mg catalyst, 3 mL benzyl alcohol and 27mL H_2_O were added in the photomicroreactor.

**Table S3.** The performance of PP-COPs in photocatalytic production of H_2_O_2_, as compared to the most advanced photocatalysts reported to date.

| **Catalyst** | **Reaction solution** | **Irradiation**  **conditions (nm)** | **H_2_O_2_ production rate (μmol g^-1^ h^-1^)** | **Ref.** |
| --- | --- | --- | --- | --- |
| PP-COP-4 | H_2_O | λ≥365 | 2758 | This work |
| PP-COP-5 | H_2_O | λ≥365 | 2507 | This work |
| PP-COP-6 | H_2_O | λ≥365 | 2256 | This work |
| PP-COP-4 | H_2_O:BA=9:1 | λ≥365 | 54488 | This work |
| PP-COP-5 | H_2_O:BA=9:1 | λ≥365 | 50526 | This work |
| PP-COP-6 | H_2_O:BA=9:1 | λ≥365 | 45430 | This work |
| TCFPP-TPD | H_2_O | λ≥420 | 1180 | [5] |
| 3P-Por-COF | H_2_O | λ>420 | 1604 | [6] |
| CTTP | H_2_O | AM 1.5 | 1850 | [7] |
| CTF-FL | H_2_O | λ>420 | 2412.1 | [8] |
| RP | H_2_O | λ>420 | 3351 | [9] |
| TBD-COF | H_2_O | λ≥400 | 5448 | [10] |
| EBBT-COF | H_2_O | λ>400 | 5686 | [11] |
| CTF-BTT | H_2_O | λ≥420 | 8099 | [12] |
| TBTN-COF | H2O | λ>420 | 11013 | [13] |
| NIES-COF-3 | H_2_O: BA=9:1 | λ≥400 | 3238.4 | [14] |
| TTA-TTTA-COF | H_2_O: Ethanol=9:1 | λ>420 | 4347 | [15] |
| PMCR-1 | H_2_O: BA=10:1 | λ>420 | 5499 | [16] |
| PAF-363 | H_2_O: Ethanol=9:1 | λ>420 | 11730 | [17] |
| PTH-SO_2_-COF | H_2_O: Methanol=9:1 | λ≥420 | 13656 | [18] |
| g-COF-DMDP-1 | H_2_O: BA=9:1 | λ>420 | 17080 | [19] |
| TTH–CTP | H_2_O: BA=9:1 | Xe lamp | 23700 | [20] |
| CTF-BTT | H_2_O: BA=9:1 | λ≥420 | 74956 | [12] |

**Section 5. Reference**

1. M. J. Frisch, G. W. Trucks, H. B. Schlegel, G. E. Scuseria, M. A. Robb, J. R. Cheeseman, G. Scalmani, V. Barone, G. A. Petersson, H. Nakatsuji, X. Li, M. Caricato, A. V. Marenich, J. Bloino, B. G. Janesko, R. Gomperts, B. Mennucci, H. P. Hratchian, J. V. Ortiz, A. F. Izmaylov, J. L. Sonnenberg, Williams, F. Ding, F. Lipparini, F. Egidi, J. Goings, B. Peng, A. Petrone, T. Henderson, D. Ranasinghe, V. G. Zakrzewski, J. Gao, N. Rega, G. Zheng, W. Liang, M. Hada, M. Ehara, K. Toyota, R. Fukuda, J. Hasegawa, M. Ishida, T. Nakajima, Y. Honda, O. Kitao, H. Nakai, T. Vreven, K. Throssell, J. A. Montgomery Jr., J. E. Peralta, F. Ogliaro, M. J. Bearpark, J. J. Heyd, E. N. Brothers, K. N. Kudin, V. N. Staroverov, T. A. Keith, R. Kobayashi, J. Normand, K. Raghavachari, A. P. Rendell, J. C. Burant, S. S. Iyengar, J. Tomasi, M. Cossi, J. M. Millam, M. Klene, C. Adamo, R. Cammi, J. W. Ochterski, R. L. Martin, K. Morokuma, O. Farkas, J. B. Foresman, D. J. Fox, Wallingford, CT **2016**.
2. A. McLean, G. Chandler, J. Contracted Gaussian basis sets for molecular calculations. I. Second row atoms, Z=11-18. *J. Chem. Phys.* **1980**, *72*, 5639.
3. S. Grimme, J. Antony, S. Ehrlich, H. Krieg, A consistent and accurate ab initio parametrization of density functional dispersion correction (DFT-D) for the 94 elements H-Pu *J. Chem. Phys.* **2010**, *132*, 154104.
4. a) W. Liao, G. Zhou, Conditions for magnetic and electronic properties of ultrathin Ni-Fe hydroxide nanosheets as catalysts: a DFT+U study. *Sci. China Mater*. **2017**, *60*, 664; b) A.V. Marenich, C.J. Cramer, D.G. Truhlar, Universal solvation model based on solute electron density and on a continuum model of the solvent defined by the bulk dielectric constant and atomic surface tensions. *J. Phys. Chem. B.* **2009**, *113*, 6378.
5. R. Zhang, H. Zhao, C. Pan, J. Zhang, L. Jian, X. Sun, R. Ji, J. Li, Y. Dong, Y. Zhu, Novel porphyrin-based donor–acceptor conjugated organic polymers for efficient photocatalytic production of hydrogen peroxide in pure water. *New J. Chem.* **2024**, *48*, 3316.
6. X. Zhan, Y. Jin, C. Qu, H. Liu, R. Jiang, Q. Zhi, D. Qi, K. Wang, B. Han, H. Pan, J. Jiang, Synergistic strategy toward enhancing photosynthesizing reactive oxygen species of covalent organic frameworks. *Adv. Funct. Mater.* **2024**, 35, 2415629.
7. Y. Guo, Y. Dong, B. Liu, B. Ni, C. Pan, J. Zhang, H. Zhao, G. Wang, Y. Zhu, Effective H_2_O_2_ photosynthesis in gas-liquid-solid triphase system with self-floating conjugated organic polymers. *Adv. Funct. Mater.* **2024**, 34, 2402920.
8. L. Zhang, C. Wang, Q. Jiang, P. Lyu, Y. Xu, Structurally locked high-crystalline covalent triazine frameworks enable remarkable overall photosynthesis of hydrogen peroxide. *J. Am. Chem. Soc*. **2024**, *146*, 29943.
9. Z. Chen, C. Chu, D, Yao, Q. Li, S. Mao, Resorcinol-phthalaldehyde resins for photosynthesis of hydrogen peroxide: modulation of electronic structure and integration of dual channel pathway. *Adv. Funct. Mater*. **2024**, 34, 2400506.
10. J. Yue, J. Luo, Z. Pan, R. Zhang, P. Yang, Q. Xu, B. Tang, Regulating the topology of covalent organic frameworks for boosting overall H_2_O_2_ photogeneration. *Angew. Chem., Int. Ed.* **2024**, 63, e202405763.
11. B. Li, J. Chen, K. Wang, D. Qi, T. Wang, J. Jiang, Ethynyl-linked donor-acceptor covalent organic framework for highly efficient photocatalytic H_2_O_2_ production. *Adv. Energy Mater.* **2025**, 2404497.
12. S. Sun, X. Yang, X. Hu, Y. Guo, Y. Zhang, C. Shu, X. Yang, H. Gao, X. Wang, I. Hussain, B. Tan, Unprecedented photocatalytic hydrogen peroxide production via covalent triazine frameworks constructed from fused building blocks. *Angew. Chem., Int. Ed.* **2024**, 64, e202416350.
13. E. Zhou, F. Wang, Y. Hui, Y. Wang, Cyanide-based ccovalent organic frameworks for enhanced overall photocatalytic hydrogen peroxide production. *Angew. Chem., Int. Ed.* **2024**, 63, e202400999.
14. D. Xue, Y. Zhang, J. Chen, H. Yang, R. Xie, S. Qi, Y. Bu, F. Liu, H. Zhang, J. Lalevée, Molecular engineering in thizolo[5,4-d]thiazole-based donor-acceptor covalent organic framework Induced high-efficient photosynthesis of H_2_O_2_. *Chem. Eng. J*. **2024**, *502*, 157874.
15. F. Tan, Y. Zheng, Z. Zhou, H. Wang, X. Dong, J. Yang, Z. Ou, H. Qi, W. Liu, Z. Zheng, X. Chen, Aqueous synthesis of covalent organic frameworks as photocatalysts for hydrogen peroxide production. *CCS Chem*. **2022**, *4*, 3751-3761.
16. P. Das. J. Roeser, A. Thomas, Solar light driven H_2_O_2_ production and selective oxidations using a covalent organic framework photocatalyst prepared by a multicomponent reaction. *Angew. Chem., Int. Ed*. **2023**, 62, e202304349.
17. L. Cao, C. Wang, H. Wang, X. Xu, X. Tao, H. Tan, G. Zhu, Rationally designed cyclooctatetrathiophene-based porous aromatic frameworks (COTh-PAFs) for efficient photocatalytic hydrogen peroxide production. *Angew. Chem., Int. Ed*. **2024**, 63, e202402095.
18. Y. Peng, L. Yuan, K. Liu, Z. Guan, S. Jin, Y. Fang, Photosynthesis of H_2_O_2_ using phenothiazine-based covalent-organic frameworks mimicking coenzyme Q. *Angew. Chem., Int. Ed*. **2024**, 64, e202423055.
19. S. Wang, Z. Xie, D. Zhu, S. Fu, Y. Wu, H. Yu, C. Lu, P, Zhou, M. Bonn, H. Wang, Q. Liao, H. Xu, X. Chen, C. Gu, Efficient photocatalytic production of hydrogen peroxide using dispersible and photoactive porous polymers. *Nat. Commun*. **2023**, *14*, 6891.
20. X. Chi, Z. Zhang, M. Li, Y. Jiao, X. Li, F. Meng, B. Xue, D. Wu, F. Zhang, Vinylene-linking of polycyclic aromatic hydrocarbons to π-extended two-dimensional covalent organic framework photocatalyst for H_2_O_2_ synthesis. *Angew. Chem., Int. Ed.* **2024**, 64, e202418895.
